# Autism-associated neuroligin 3 deficiency in medial septum causes social deficits and sleep loss in mice

**DOI:** 10.1172/JCI176770

**Published:** 2024-07-26

**Authors:** Haiyan Sun, Yu Shen, Pengtao Ni, Xin Liu, Yan Li, Zhentong Qiu, Jiawen Su, Yihan Wang, Miao Wu, Xiangxi Kong, Jun-Li Cao, Wei Xie, Shuming An

**Affiliations:** 1 Jiangsu Province Key Laboratory of Anesthesiology and Jiangsu Province Key Laboratory of Anesthesia and Analgesia Application Technology, NMPA Key Laboratory for Research and Evaluation of Narcotic and Psychotropic Drugs, Xuzhou Medical University, Xuzhou, China.; 2 The Key Laboratory of Developmental Genes and Human Disease, Ministry of Education, School of Life Science and Technology, Southeast University, Nanjing, China.; 3 Department of Anesthesiology, Affiliated Hospital of Xuzhou Medical University, Xuzhou, China.

**Keywords:** Neuroscience, Behavior, Neurological disorders

## Abstract

Patients with autism spectrum disorder (ASD) frequently experience sleep disturbance. Genetic mutations in the neuroligin 3 (*NLG3*) gene are highly correlative with ASD and sleep disturbance. However, the cellular and neural circuit bases of this correlation remain elusive. Here, we found that the conditional knockout of *Nlg3* (*Nlg3*-CKO) in the medial septum (MS) impairs social memory and reduces sleep. *Nlg3* CKO in the MS caused hyperactivity of MS^GABA^ neurons during social avoidance and wakefulness. Activation of MS^GABA^ neurons induced social memory deficits and sleep loss in C57BL/6J mice. In contrast, inactivation of these neurons ameliorated social memory deficits and sleep loss in *Nlg3*-CKO mice. Sleep deprivation led to social memory deficits, while social isolation caused sleep loss, both resulting in a reduction in NLG3 expression and an increase in activity of GABAergic neurons in the MS from C57BL/6J mice. Furthermore, MS^GABA^-innervated CA2 neurons specifically regulated social memory without impacting sleep, whereas MS^GABA^-innervating neurons in the preoptic area selectively controlled sleep without affecting social behavior. Together, these findings demonstrate that the hyperactive MS^GABA^ neurons impair social memory and disrupt sleep resulting from *Nlg3* CKO in the MS, and achieve the modality specificity through their divergent downstream targets.

## Abstract 2

Autism-associated neuroligin-3 deficiency in medial septum causes social deficits and sleep loss through divergent downstream circuit targets.

## Graphical abstract

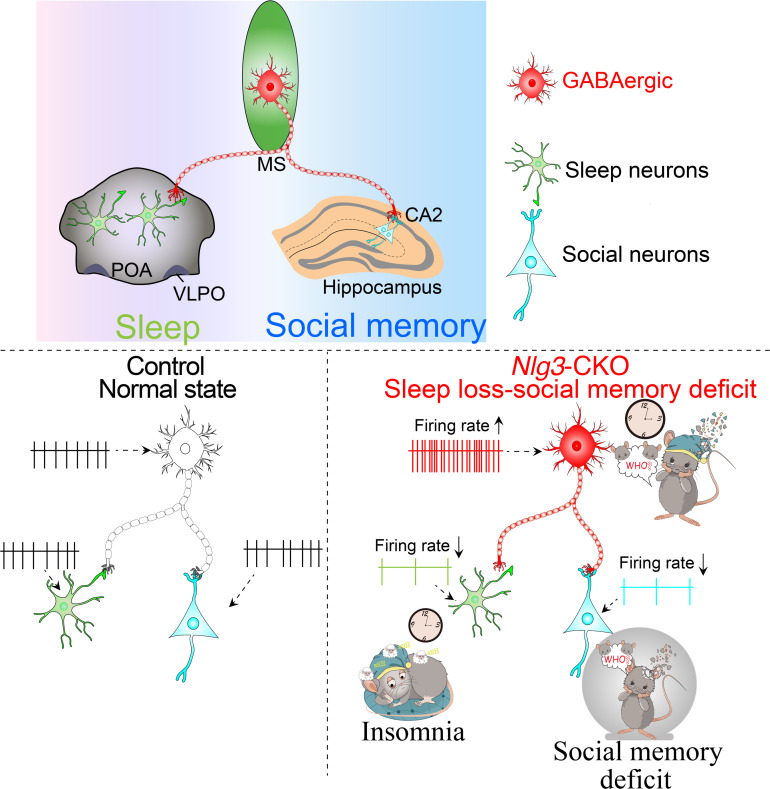

## Introduction

Clinically, up to 80% of patients with autism spectrum disorder (ASD) suffer from sleep disturbances (1). This relationship between ASD and sleep disturbances is bidirectional, meaning that the worsening of sleep problems can exacerbate the core symptoms of ASD, and vice versa (2–4). Specifically, sleep deprivation leads to social withdrawal in humans (5) and social memory deficits in rodents (6). On the other hand, chronic social isolation has been shown to result in sleep impairments in flies, rodents, and humans (7–9). However, the neural circuit mechanism underlying this relationship remains poorly understood, impeding the progress of efficacious clinical interventions.

The medial septum (MS) is involved in not only the modulation of social memory, but also sleep-wake regulation. Notably, chemogenetic manipulation of MS neurons bidirectionally regulates social memory (10). In parallel, manipulating MS glutamatergic neurons bidirectionally modulates sleep-wakefulness (11). These raise the question of whether the same MS neurons simultaneously regulate both social memory and sleep, despite these 2 processes belonging to 2 distinct physiological modalities.

Neuroligin 3 (NLG3) is a cell adhesion molecule located postsynaptically, playing a role in synaptogenesis and synaptic transmission (12–14). Mice with complete knockout of *Nlg3* display social memory impairments, which are similar to those observed in autistic patients (15–17). Additionally, *Nlg3*-knockout rats exhibit reduced duration of non–rapid eye movement (NREM) sleep (18). Nevertheless, it is uncertain whether conditional knockout of *Nlg3* (*Nlg3*-CKO) in the MS causes impairments both in social and sleep behaviors.

Our current study aimed to investigate how the hyperactivity of MS neurons, following *Nlg3* CKO in the MS, leads to sleep and social impairments through distinct downstream circuit targets. By combining optrode recording, viral tracing, fluorescence in situ hybridization (FISH), and optogenetic manipulation, we detected a dual-encoding ensemble (MS^GABA^ neurons) in *Nlg3*-CKO mice. These neurons exhibited hyperactivity during either social avoidance or wakefulness. Activation of MS^GABA^ neurons impaired social memory and reduced NREM sleep, while inactivation of these neurons ameliorated the deficits in both social memory and NREM sleep. Both sleep deprivation and social isolation reduced NLG3 expression and increased MS^GABA^ neuron activity. We have further determined that CA2 neurons, which receive input from MS^GABA^ neurons, specifically regulated social memory without impacting sleep, whereas MS^GABA^-innervating neurons in the preoptic area selectively regulated sleep without affecting social behavior. These results demonstrate that MS^GABA^ neurons impair social memory and disrupt sleep following *Nlg3* CKO in the MS, and achieve the modality specificity of social memory versus sleep through their distinct downstream projections.

## Results

### CKO of Nlg3 in the MS impairs social memory and reduces sleep.

**Figure 1:**
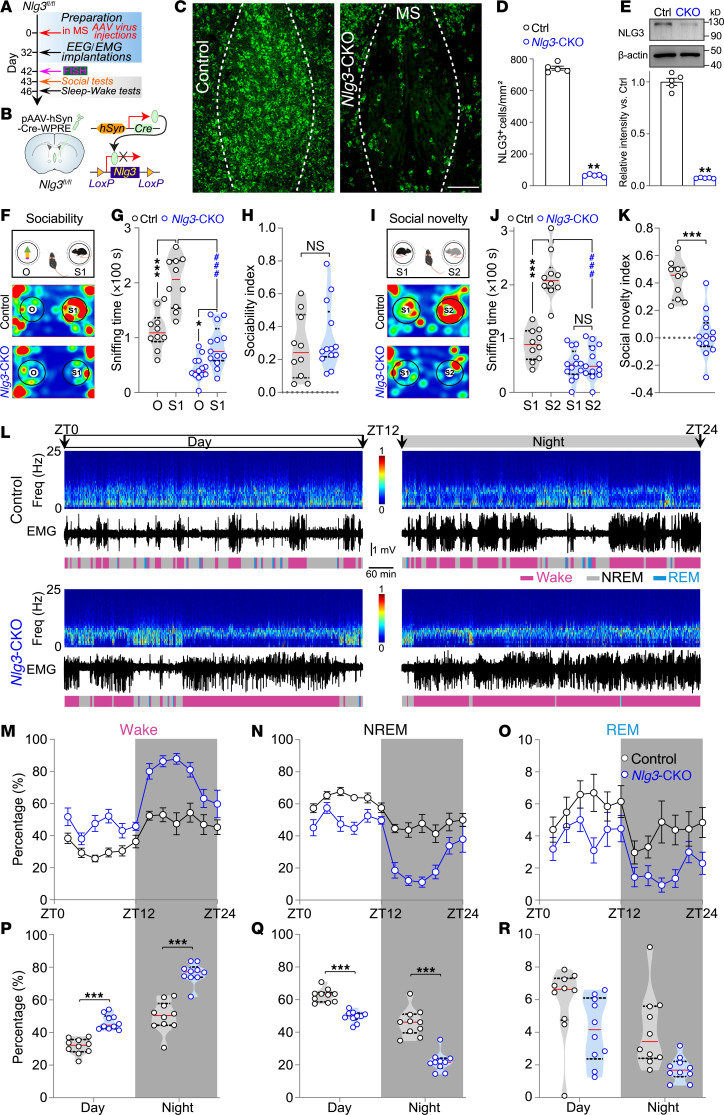
Conditional knockout of *Nlg3* in the MS impairs social memory and reduces sleep. (**A**) Schematic of the experimental procedure. (**B**) pAAV-hSyn-Cre-WPRE was injected into the MS of *Nlg3^fl/fl^* mice to conditionally knock out *Nlg3* (*Nlg3*-CKO mice). (**C**–**E**) Representative FISH images (**C**) and quantification of NLG3 mRNA and protein in MS between control and *Nlg3*-CKO mice 6 weeks after virus injection (**D** and **E**, mean ± SEM, *n* = 5 mice). Scale bar: 200 μm. (**F**–**H**) Representative heatmaps (**F**) and quantification of sniffing time and sociability index between control (*n* = 10 mice) and *Nlg3*-CKO mice (*n* = 13 mice) during the sociability test (**G**, *F*_[1,__21]_ = 5.71; and **H**, *P* = 0.483). In this and the following violin plots, data are presented as median (red line) with 25th and 75th percentiles (dashed line). (**I**–**K**) Representative heatmaps (**I**) and quantification of sniffing time and social novelty index between control and *Nlg3*-CKO mice during the social novelty test (**J**, *F*_[1,__21]_ = 76.6; and **K**). (**L**) Twenty-four hours of continuous EEG spectrogram, EMG trace, and brain states (color-coded). ZT, zeitgeber time. (**M**–**O**) The graphs illustrate the average (mean ± SEM) percentages of wake, NREM, or REM sleep during the day and night for control (*n* = 10 mice) and *Nlg3*-CKO mice (*n* = 10 mice). (**P**–**R**) *Nlg3*-CKO mice exhibited significantly more wakefulness (*F*_[1,__18]_ = 10.63), significantly less NREM (*F*_[1,__18]_ = 10.88), and a nonsignificant difference in REM sleep (*F*_[1,__18]_ = 0.403, *P* = 0.534) during the day and night. *P < 0.05; **P < 0.01; ***P < 0.001; ^###^*P* < 0.001 by 2-tailed Mann-Whitney test (**D**, **E**, **H**, and **K**) or 2-way ANOVA with Bonferroni’s post hoc test (**G**, **J**, and **P**–**R**). NS, not significant. See the Supporting Data Values file for statistical details.

Sleep disturbances and social deficits are commonly observed in patients with ASD (1). The MS has been implicated in regulating sleep-wake and social behaviors (10, 11). To investigate the potential role(s) of autism-associated NLG3 in the MS related to sleep-wake and social behaviors, we generated CKO mice by injecting adeno-associated virus (pAAV-hSyn-Cre) into the MS of *Nlg3^fl/fl^* mice (Figure 1, A and B), resulting in the specific deletion of *Nlg3* in the MS (*Nlg3*-CKO mice). Six weeks after virus injection, we assessed the mRNA and protein levels of NLG3 using FISH, real-time reverse transcription polymerase chain reaction (RT-PCR), and Western blotting, respectively. Both the mRNA and protein levels of NLG3 were significantly reduced in the MS of *Nlg3*-CKO mice (Figure 1, C–E, and Supplemental Figure 1; supplemental material available online with this article; https://doi.org/10.1172/JCI176770DS1).

Social behavior and social memory were assessed in modified 3-chamber tests of sociability and social novelty, respectively. Sociability was initially tested for the subject mouse in a partner-object trial, where it was paired with a partner (stranger 1, S1) in one cage and an object (O) in the other cage (Figure 1F). Both control and *Nlg3*-CKO mice spent more time sniffing S1 compared with the object (Figure 1, F and G). *Nlg3*-CKO mice spent significantly less time sniffing S1 in comparison with the control mice (Figure 1G). However, both groups of mice exhibited a similar sociability index (Figure 1H), suggesting normal sociability in *Nlg3*-CKO mice. In the social novelty test, another unfamiliar mouse was introduced as a novel social partner (stranger 2, S2) to replace the object (Figure 1I). The control mice showed a preference for S2, with a normal social novelty index, whereas *Nlg3*-CKO mice displayed no clear preference for S2 and had a significantly lower social novelty index (Figure 1, J and K). *Nlg3*-CKO and control mice travelled similar distances in 3-chamber tests (Supplemental Figure 2A). These findings suggest that NLG3 deficiency specifically in the MS results in an impairment of social memory.

To assess sleep-wake amounts in *Nlg3*-CKO mice, we implanted cortical electroencephalographic (EEG) and nuchal electromyographic (EMG) electrodes, followed by conducting 24-hour baseline home-cage sleep recordings (Figure 1L). *Nlg3*-CKO mice exhibited a notable reduction in NREM and a partial decrease in rapid eye movement (REM) sleep, along with a complementary increase in wakefulness as compared with the control mice (Figure 1, M–R). Thus, social memory deficits and sleep disturbance coexisted in *Nlg3*-CKO mice.

To investigate whether NLG3 deletion in the MS influences social interaction, sucrose preference, basal locomotion, anxiety-like behaviors, and cognitive ability for novel objects, we performed a battery of behavioral tests. In the social interaction test, *Nlg3*-CKO mice spent significantly less time in anogenital sniffing and pursuit than the control mice (Supplemental Figure 2, B and C). In the sucrose preference test, *Nlg3*-CKO and control mice exhibited similar sucrose preferences (Supplemental Figure 2D). Additionally, *Nlg3*-CKO mice showed normal locomotor activity and anxiety-like behaviors in the open field and elevated plus maze tests (Supplemental Figure 2, E–M), respectively. In the novel object recognition test, which assesses novel object recognition memory, both *Nlg3*-CKO and control mice showed comparable preference for the novel object (Supplemental Figure 2, N–S). These findings suggest that *Nlg3*-CKO mice have normal levels of locomotion, anxiety-like behaviors, sucrose preference, and object recognition memory, but deficits in social interaction.

Together, these findings reveal that CKO of *Nlg3* in the MS impairs social interaction behaviors and reduces sleep.

### Nlg3 knockout in the MS causes hyperactivity of MS^GABA^ neurons during social avoidance and wakefulness.

**Figure 2:**
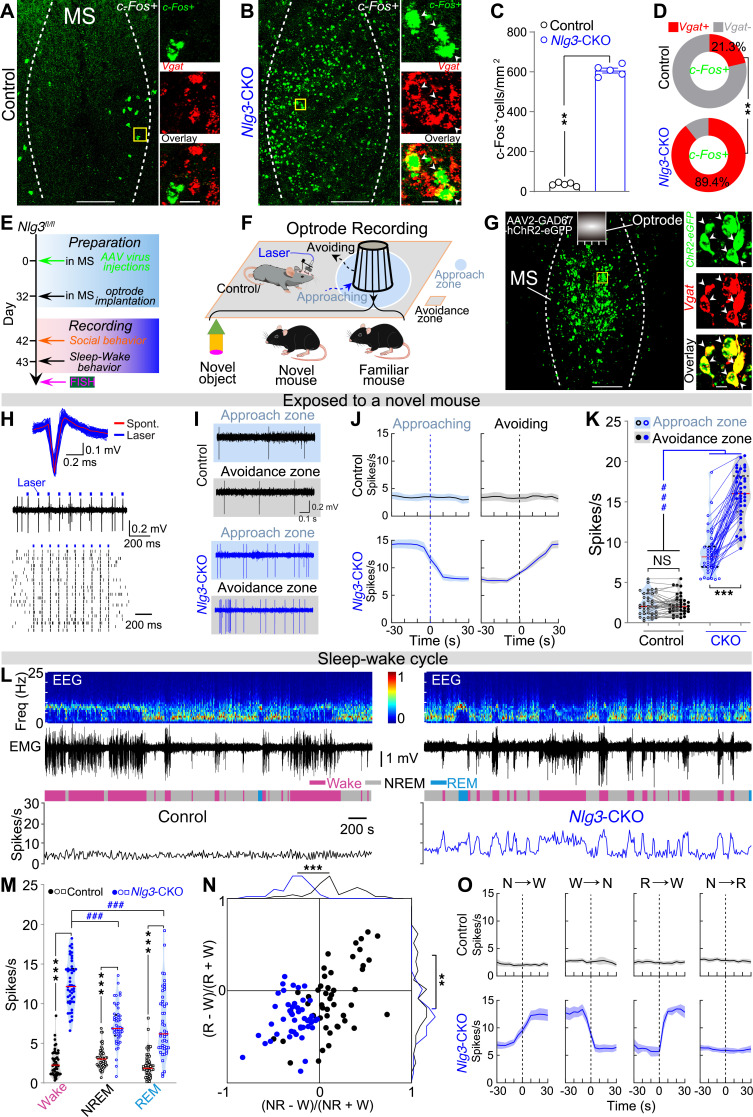
*Nlg3* knockout in the MS causes hyperactivity of MS^GABA^ neurons during social avoidance and wakefulness. (**A**–**C**) Representative FISH images (**A** and **B**) and quantification of c-Fos expression in MS between control and *Nlg3*-CKO mice (**C**, *n* = 5 mice). Scale bars: 200 μm (left) and 20 μm (right). (**D**) Quantification of the overlap between c-Fos^+^ cells and *Vgat* (*n* = 5 mice). (**E** and **F**) Schematic diagram for experimental protocols and optrode recording. (**G**) Representative images of the MS from an *Nlg3*-CKO mouse. Scale bars: 200 μm (left) and 20 μm (right). (**H**) Optogenetic tagging and identification of MS^GABA^ neuron. Waveforms (top), a representative raw trace (middle), and raster plots (down) from an identified MS^GABA^ neuron. (**I**–**K**) Example recording of spontaneous spikes (**I**) and mean firing rates (**J**, shading represents ±SEM) and quantification of the identified MS^GABA^ neurons from control and *Nlg3*-CKO mice (**K**, *n* = 38 units from 7 mice, *F*_[1,__74]_ = 157.9) during the approach and avoidance of a novel mouse. (**L** and **M**) Representative trace of firing rate from a MS^GABA^ neuron (**L**) and quantification of the identified MS^GABA^ neurons in control mice and *Nlg3*-CKO mice (**M**, *n* = 48 units from 9 mice, *F*_[2,__188]_ = 59.83) during wakefulness, NREM, and REM sleep. (**N**) Firing rate modulation of identified MS^GABA^ neuron from both control and *Nlg3*-CKO mice. W, wake; R, REM; NR, NREM. (**O**) Mean firing rates of identified MS^GABA^ neurons from both mice during different brain state transitions. Shading represents ±SEM. **P < 0.01; ***P < 0.001; ^###^*P* < 0.001 by 2-tailed Mann-Whitney test (**C** and **N**) or 2-way ANOVA with Bonferroni’s post hoc test (**K** and **M**). NS, not significant.

c-Fos has been widely recognized as a reliable indicator of neuronal activity (19). In order to explore the potential activation of neurons in the MS resulting from the CKO of *Nlg3*, we conducted a FISH experiment for *c-fos* mRNA. The levels of *c-fos* mRNA in the MS of *Nlg3*-CKO mice were found to be significantly higher than those in the control mice (Figure 2, A–C). Additionally, we performed double FISH of *c-Fos* with *Vgat*. The majority of *c-fos*–expressing neurons in the MS of *Nlg3*-CKO mice coexpressed *Vgat*, whereas a considerably smaller proportion of neurons in the MS of control mice expressed *Vgat* (Figure 2D). These results show that the majority of active neurons are GABAergic neurons following *Nlg3* CKO in the MS.

We next wondered how these neurons behaved during social and sleep-wake behaviors. To address this question, we first labeled these neurons with channelrhodopsin-2 (ChR2) by injecting an AAV vector (AAV2-GAD67-hChR2-eGFP) into the MS of both control and *Nlg3*-CKO mice (Figure 2, E–G, and Supplemental Figure 3A). As expected, AAV expressing ChR2 fused with the enhanced green fluorescent protein (eGFP) selectively labeled GABAergic neurons in the MS (Figure 2G). Subsequently, we performed optogenetic tagging and optrode recordings (11, 20) to monitor ChR2-expressing GABAergic neurons in the MS of control and *Nlg3*-CKO mice during social behaviors and the sleep-wakefulness cycles (Figure 2, E–G). Optogenetic stimulations (473 nm, 1-ms pulse duration, 10 Hz × 1 second) were applied to screen and define the ChR2-expressing GABAergic neurons according to the following 3 criteria: (a) laser stimulations should evoke spikes reliably (>70% of firing rate), (b) laser-evoked spikes should have short latencies (1–3 ms) and low jitters (<3 ms) to rule out network implication, and (c) laser-evoked and spontaneous spikes should display similar waveforms (Figure 2H and Supplemental Figure 3, B–D).

To monitor the activity of MS^GABA^ neurons in both *Nlg3*-CKO and control mice during object and social exploration, we recorded MS^GABA^ neuron activity while the subject mouse was approaching and avoiding a novel inanimate object, a novel mouse, as well as the same, now familiar mouse. Notably, the activity in MS^GABA^ neurons of *Nlg3*-CKO mice decreased significantly when approaching a novel mouse, but increased remarkably when avoiding the same mouse (Figure 2, I–K). No significant changes were detected in MS^GABA^ neuron activity of control mice during the approach and avoidance of a novel mouse. Additionally, the firing rate of MS^GABA^ neurons in *Nlg3*-CKO mice was significantly higher when the mice were in the “avoidance zone” than in the “approach zone,” and also higher than that of control mice (Figure 2K). However, the activity of MS^GABA^ neurons did not change appreciably in both *Nlg3*-CKO and control mice when they approached and avoided a novel object or the familiar mouse (Supplemental Figure 4, A–F). Notably, the firing rate of MS^GABA^ neurons in *Nlg3*-CKO mice was found to be higher than that of control mice (Supplemental Figure 4, B and E). Moreover, no significant changes were observed in the firing rate of unidentified MS neurons from *Nlg3*-CKO mice during social approach-avoidance of a novel mouse (Supplemental Figure 4, G–I). These findings suggest that MS^GABA^ neurons of *Nlg3*-CKO mice exhibited increased activities in response to the avoidance of a novel mouse.

Subsequently, we recorded the activity of these MS^GABA^ neurons in both mice across sleep and wakefulness (Figure 2L). In *Nlg3*-CKO mice, the average firing rate of MS^GABA^ neurons was considerably higher during wakefulness in comparison with NREM and REM sleep (Figure 2, L and M). Furthermore, the firing rate of these neurons in *Nlg3*-CKO mice was significantly higher than that in control mice during wakefulness, NREM, and REM sleep (Figure 2M). In contrast with the MS^GABA^ neurons in *Nlg3*-CKO mice, the MS^GABA^ neurons from control mice exhibited similar mean firing rates across awake, NREM, and REM states (Figure 2, L and M).

We further quantified the brain-state preference of recorded neurons by calculating their NREM-wake modulation [(*R*_NREM_ – *R*_wake_)/(*R*_NREM_ + *R*_wake_)], where *R* represents the average firing rate within each brain state and REM-wake modulation [(*R*_REM_ – *R*_wake_)/(*R*_REM_ + *R*_wake_)] (Figure 2N). In comparison with the MS^GABA^ neurons from control mice, the MS^GABA^ neurons in *Nlg3*-CKO mice exhibited reduced NREM-wake and REM-wake modulations. Additional analysis was conducted on MS^GABA^ neurons in both mice during the transitions between 2 distinct brain states. Specifically, during transitions from NREM or REM sleep to wakefulness in *Nlg3*-CKO mice, the averaged firing rate of these neurons increased sharply, whereas the high firing rate of these neurons gradually decreased during transitions from wakefulness to NREM sleep (Figure 2O). In contrast, no such activity change was observed in MS^GABA^ neurons from control mice. Therefore, the increased activity of MS^GABA^ neurons from *Nlg3*-CKO mice during wakefulness may contribute to the reduced NREM sleep and increased wakefulness.

Taken together, these results indicate that *Nlg3* CKO in the MS leads to hyperactivity of MS^GABA^ neurons during social avoidance and wakefulness, suggesting a potential relationship between the increased MS^GABA^ neuron activity and social memory impairments as well as sleep loss.

### Activation of MS^GABA^ neurons induces social memory deficits and sleep loss in C57BL/6J mice.

**Figure 3:**
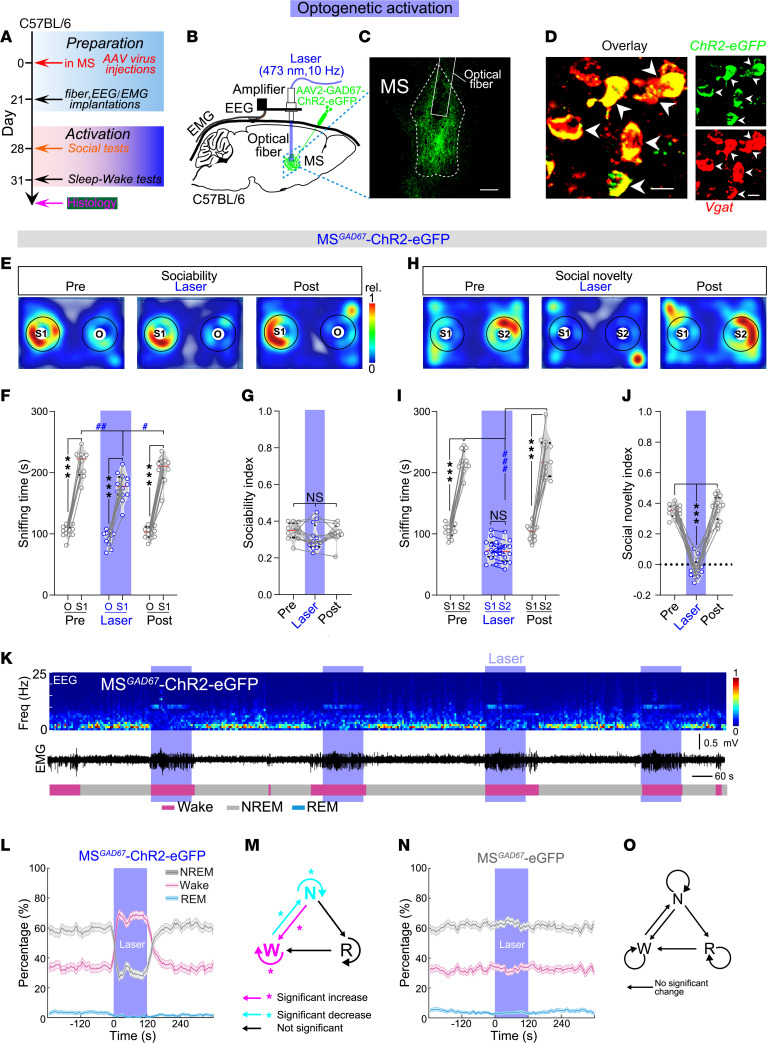
Activating MS^GABA^ neurons induces social memory deficits and sleep loss in C57BL/6J mice. (**A**) Schematic diagram of the experimental procedure. (**B**) AAV2-GAD67-hChR2-eGFP was injected into the MS and blue laser light (473 nm, 10 Hz) was applied to the MS. (**C**) Representative image showing selective transduction of hChR2-eGFP in the MS where an optical fiber is located. Scale bar: 200 μm. (**D**) Representative images showing hChR2-eGFP–expressing neurons colocalized with Vgat. Scale bar: 20 μm. (**E**–**G**) Representative heatmaps (**E**) and quantification of sniffing time (**F**; *n* = 12 mice, *F*_[2,__33]_ = 5.811) and sociability index (**G**, *F*_[1.26,__13.9]_ = 2.692) in MS^GAD67^-ChR2 mice during the sociability test. (**H**–**J**) Representative heatmaps (**H**) and quantification of sniffing time (**I**; *F*_[2,__33]_ = 116.8) and social novelty index (**J**, *F*_[1.74,__19.13]_ = 132.3) in MS^GAD67^-ChR2 mice during the social novelty test. (**K**) Representative EEG spectrogram (top), EMG trace (middle), and brain states (bottom) from a MS^GAD67^-ChR2 mouse. Blue stripe indicates laser stimulation (473 nm, 10 Hz, 120 seconds). (**L**) Percentage of time in different brain states before, during, and after blue laser (473 nm, 10 Hz, 120 seconds) activation of MS^GABA^ neurons (*n* = 12 mice, NREM, REM, and Wake). Shading represents ±SEM. (**M**) The changes in transition probability between each pair of brain states in MS^GAD67^-ChR2 mice during blue laser stimulation. (**N** and **O**) No significant change in percentage of time in different brain states (**N**, *n* = 10 mice, NREM, *P* = 0.189; wake, *P* = 0.196; REM, *P* = 0.13) and transition probability (**O**, *P* > 0.05) in MS^GAD67^-eGFP control mice. *P < 0.05; ***P < 0.001; ^#^*P* < 0.05; ^##^*P* < 0.01; ^###^*P* < 0.001 by 2-way (**F** and **I**) or 1-way (**G** and **J**) repeated-measures ANOVA with Bonferroni’s post hoc test, or bootstrap test (**L**–**O**). NS, not significant.

The above correlative results prompted us to explore the potential causal roles of hyperactivity in MS^GABA^ neurons in regulating social behavior and sleep/wakefulness. Subsequently, we investigated whether the hyperactivity of MS^GABA^ neurons could be responsible for the observed social memory impairment and sleep loss (Figure 3A). In C57BL/6J mice, we selectively expressed ChR2 in MS^GABA^ neurons by injecting AAV2-GAD67-hChR2-eGFP (hereafter referred to as MS^GAD67^-ChR2 mice, Supplemental Figure 5A). The MS^GABA^ neurons were optogenetically activated using a blue laser (473 nm) at 10 Hz (Figure 3, B–D). During the sociability test, the consistent activation of MS^GABA^ neurons significantly reduced the sniffing time on S1, but did not affect the sociability index in MS^GAD67^-ChR2 mice (Figure 3, E–G). During the social novelty test, the persistent activation of MS^GABA^ neurons led to a significant decrease in sniffing time on S2 and social novelty index in MS^GAD67^-ChR2 mice (Figure 3, H–J). In the MS^GAD67^-eGFP control mice, in which AAV2-GAD67-eGFP was injected into the MS of C57BL/6J mice, the same blue laser stimulation had no effect on sociability and social memory (Supplemental Figure 5, B–G). These results indicate that optogenetic activation of MS^GABA^ neurons causes impairments in social memory.

On the other hand, we applied the same 10 Hz blue laser stimulations to MS^GABA^ neurons in the regulation of sleep-wake. Optical activation of MS^GABA^ neurons induced immediate transitions from NREM sleep to wakefulness (Figure 3K), a notable reduction in NREM and REM sleep, and a complementary increase in wakefulness (Figure 3L). We further quantified the changes in transition probability between each pair of brain states during the activation of MS^GABA^ neurons. A significant increase was observed in the NREM→wake and wake→wake transitions during the activation (Figure 3M and Supplemental Figure 5H). Conversely, a complementary decrease was detected in the wake→NREM and NREM→NREM transitions in MS^GAD67^-ChR2 mice, indicating an increase in both initiation and maintenance of wakefulness, as well as a reduction in NREM sleep. However, no significant changes in brain state percentage and transition probability were found in MS^GAD67^-eGFP control mice during laser stimulation (Figure 3, N and O, and Supplemental Figure 5I). Collectively, these findings demonstrate that the activation of MS^GABA^ neurons induces social memory deficits and sleep loss in C57BL/6J mice.

### Both sleep deprivation and social isolation reduce NLG3 expression and increase MS^GABA^ neuron activity.

**Figure 4:**
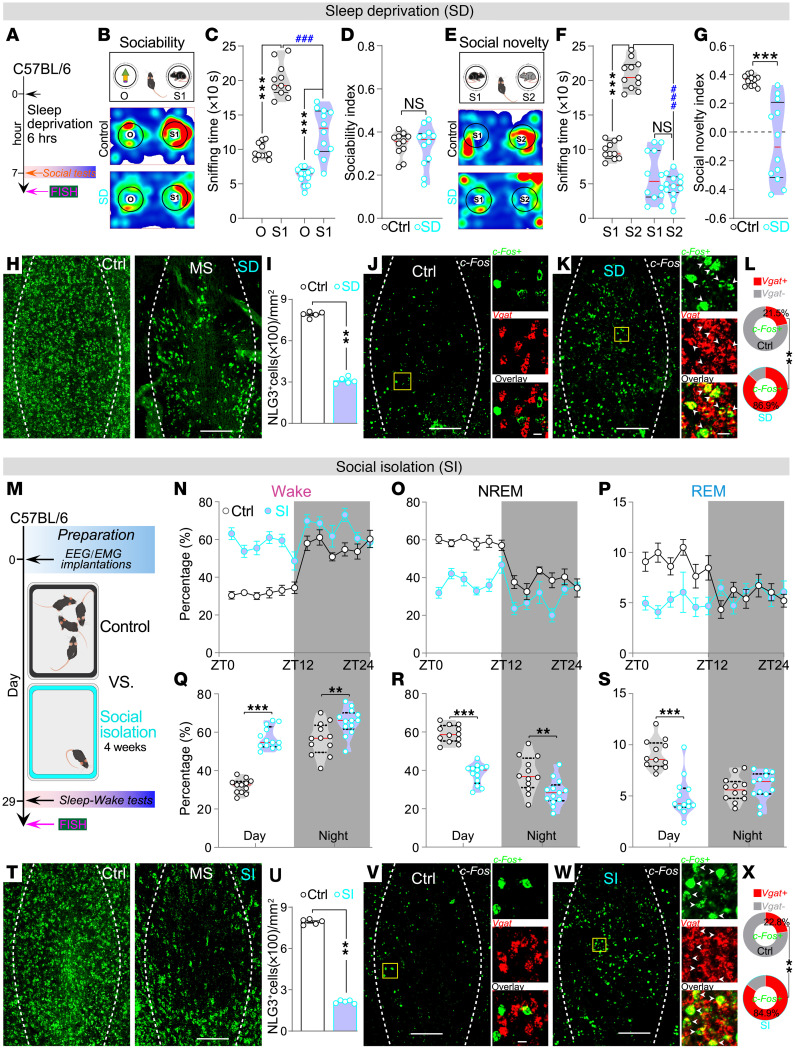
Both sleep deprivation and social isolation reduce NLG3 expression and increase MS^GABA^ neuron activity. (**A**) Experimental scheme. (**B**) Representative heatmaps showing occupancy time in a control and sleep-deprived (SD) mouse during sociability test. (**C** and **D**) Quantification of sniffing time (**C**, control, *n* = 11 mice; SD, *n* = 12 mice; *F*_[1,__21]_ = 14.52) and sociability index (**D**, *P* = 0.833) in the sociability test. (**E**) Representative heatmaps of occupancy time during social novelty test from a control and SD mouse. (**F** and **G**) Quantification of sniffing time (**F**, *F*_[1,__21]_ = 128.8) and social novelty index (**G**) in the social novelty test. (**H** and **I**) Representative FISH images (**H**) and quantification of *Nlg3* mRNA in the MS between control and SD mice (**I**, *n* = 5 mice). (**J**–**L**) Representative FISH images (**J** and **K**) showing coexpression of *c-fos* and *Vgat* mRNA in the MS, and quantification between control and SD mice (**L**, *n* = 5 mice). Scale bars: 200 μm (left) and 20 μm (right). (**M**) Schematic showing experiment protocol. (**N**–**P**) The average (mean ± SEM) percentages of wake, NREM, or REM sleep during the day and night for control (*n* = 12 mice) and socially isolated (SI) mice (*n* = 13 mice). ZT, zeitgeber time. (**Q**–**S**) SI mice exhibited significantly more wakefulness (*F*_[1,__23]_ = 21.98), less NREM (*F*_[1,__23]_ = 9.567), and REM sleep (*F*_[1,__23]_ = 20.37) during the day and night. (**T** and **U**) Representative FISH images (**T**) and quantification of *Nlg3* mRNA in the MS between control and SI mice (**U**, *n* = 5 mice). (**V**–**X**) Representative FISH images (**V** and **W**) showing coexpression of *c-fos* and *Vgat* mRNA in the MS and quantification between control and SI mice (**X**, *n* = 5 mice). *P < 0.05; **P < 0.01; ***P < 0.001 by 2-way ANOVA with Bonferroni’s post hoc test (**C**, **F**, and **Q**–**S**) or 2-tailed Mann-Whitney test (**D**, **G**, **I**, **L**, **U**, and **X**).

We next asked whether direct sleep deprivation and social isolation could mimic the effects of optogenetic activation of MS^GABA^ neurons to regulate sleep and social behaviors. To address this question, we performed a 6-hour sleep deprivation in C57BL/6J mice (Figure 4A). This sleep deprivation induced deficits in social memory (Figure 4, B–G), with effects similar to that resulting from optogenetic activation of MS^GABA^ neurons. Furthermore, we checked the mRNA level of NLG3 and c-Fos. There was a significantly lower level of *Nlg3* mRNA in MS of mice subjected to sleep deprivation than in control mice (Figure 4, H and I). Moreover, the majority of *c-fos*–expressing neurons in the MS of sleep-deprived mice coexpressed *Vgat* mRNA (Figure 4, J–L), which is consistent with high colocalization of *c-fos* and *Vgat* mRNA in the MS of *Nlg3*-CKO mice (Figure 2, A–D).

On the other hand, we performed 4 weeks of social isolation in C57BL/6J mice (Figure 4M). Socially isolated mice exhibited considerably less NREM sleep and more wakefulness than control (group housed) mice (Figure 4, N–S). Similarly, the mRNA level of *Nlg3* in the MS from socially isolated mice was consistently lower than that from control mice (Figure 4, T and U). Additionally, most of the MS neurons expressing *c-fos* in socially isolated mice also coexpressed *Vgat* (Figure 4, V–X). Collectively, these findings demonstrate that sleep deprivation impairs social memory and chronic social isolation reduces sleep, implicating potential roles of NLG3 protein and MS^GABA^ neurons in social and sleep behaviors.

### Inactivation of MS^GABA^ neurons increases NREM sleep and ameliorates social memory deficits in Nlg3-CKO mice.

**Figure 5:**
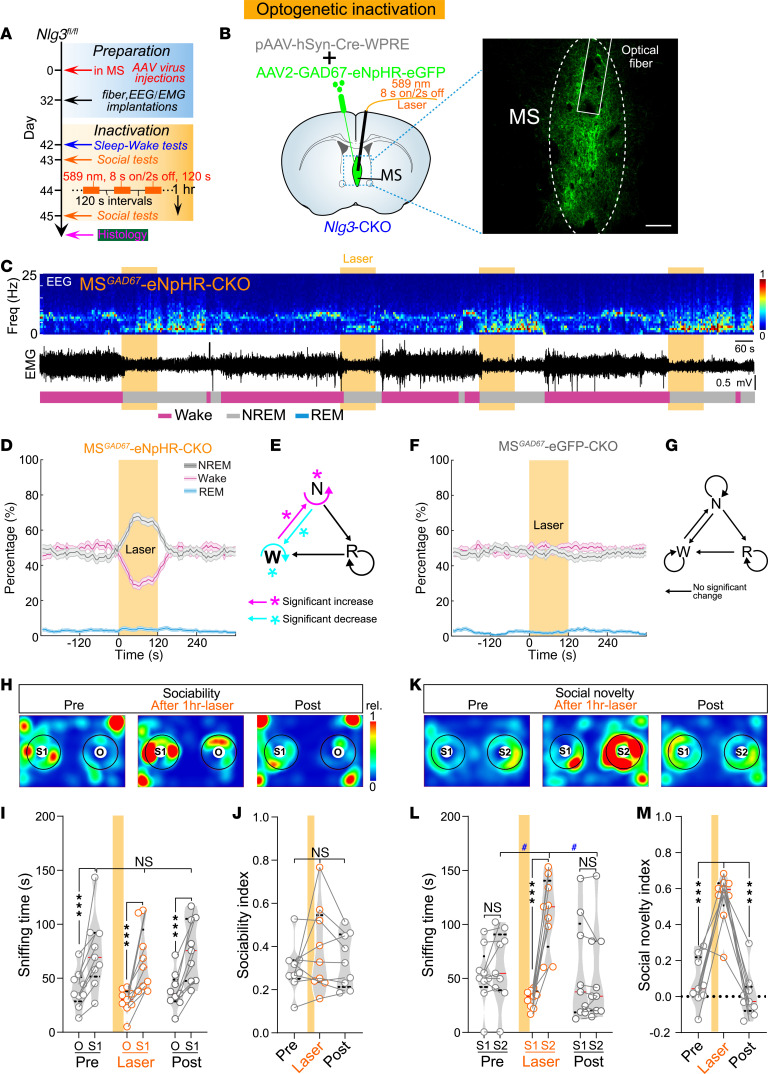
Inactivating MS^GABA^ neurons ameliorates sleep loss and social memory deficits in *Nlg3*-CKO mice. (**A**) Schematic diagram of the experimental procedure. (**B**) Expression of eNpHR-eGFP in MS^GABA^ neurons of *Nlg3*-CKO mice (MS^GAD67^-eNpHR-CKO mice). Scale bar: 200 μm. (**C**) Baseline conditions and yellow laser inactivation of MS^GABA^ neurons from an MS^GAD67^-eNpHR-CKO mouse with representative EEG spectrogram (top), EMG trace (middle), and brain states (bottom). Yellow stripe indicates laser stimulation (589 nm, yellow laser, 8 seconds on/2 seconds off, 120 seconds). (**D**) Percentage of time in different brain states before, during, and after yellow laser inactivation of MS^GABA^ neurons from MS^GAD67^-eNpHR-CKO mice (*n* = 9 mice, NREM and Wake; REM, *P* = 0.252). Shading represents ±SEM. (**E**) The changes in transition probability between each pair of brain states in MS^GAD67^-eNpHR-CKO mice during yellow laser stimulation. (**F** and **G**) No significant change in percentage of time in different brain states (**F**, *n* = 8 mice, NREM, *P* = 0.25; wake, *P* = 0.154; REM, *P* = 0.174) and transition probability (**G**, *P* > 0.05) in MS^GAD67^-eGFP-CKO control mice. (**H**–**J**) Representative heatmaps (**H**) and quantification of sniffing time (**I**; *n* = 9 mice, *F*_[2,__24]_ = 0.051, *P* = 0.951) and sociability index (**J**, *F*_[1.27,__10.17]_ = 1.719*, P* = 0.224) in MS^GAD67^-eNpHR-CKO mice during the sociability test. (**K**–**M**) Representative heatmaps (**K**) and quantification of sniffing time (**L**; *F*_[2,__24]_ = 38.29) and social novelty index (**M**, *F*_[1.896,__15.17]_ = 39.65) in MS^GAD67^-eNpHR-CKO mice during the social novelty test. *P < 0.05; ***P < 0.001; ^#^*P* < 0.05 by bootstrap test (**D**–**G**), or 2-way (**I** and **L**) or 1-way (**J** and **M**) repeated-measures ANOVA with Bonferroni’s post hoc test. NS, not significant.

In contrast with activation, we next sought to determine whether the inactivation of MS^GABA^ neurons could ameliorate sleep loss and social memory deficits in *Nlg3*-CKO mice. Specifically, we injected mixed viruses (pAAV-hSyn-Cre and AAV2-GAD67-eNpHR-eGFP) into the MS of *Nlg3^fl/fl^* mice (MS^GAD67^-eNpHR-CKO mice; Figure 5, A and B, and Supplemental Figure 6I). Six weeks after virus injection, optogenetic inhibition (589 nm, 8 seconds on/2 seconds off, 2 minutes) of MS^GABA^ neurons induced a significant increase in NREM sleep, with a consequent reduction in wakefulness in *Nlg3*-CKO mice (Figure 5, C–E). Further analyses reveal that these changes were attributable to the increased NREM→NREM and wake→NREM transitions, as well as the decreased wake→wake and NREM→wake transitions in MS^GAD67^-eNpHR-CKO mice (Figure 5E and Supplemental Figure 6A). In the MS^GAD67^-eGFP-CKO control mice, in which pAAV-hSyn-Cre and AAV2-GAD67-eGFP were injected into the MS of *Nlg3^fl/fl^* mice, the optogenetic inhibition had no discernible effect on either the brain state percentages or the transition probability (Figure 5, F and G, and Supplemental Figure 6B).

Moreover, silencing MS^GABA^ neurons in MS^GAD67^-eNpHR-CKO mice significantly decreased the sniffing time and total distance travelled in sociability and social novelty tests (Supplemental Figure 6, C–H). These results may be due to a significant increase in NREM sleep induced by inhibition of MS^GABA^ neurons. Therefore, to avoid dozing off or falling asleep in MS^GAD67^-eNpHR-CKO mice during social tests, we applied a repetitive yellow laser stimulation paradigm (589 nm, 8 seconds on/2 seconds off, 120 seconds) that inhibited MS^GABA^ neurons for 1 hour prior to the social tests (Figure 5A). After application of yellow laser stimulation, MS^GAD67^-eNpHR-CKO mice exhibited no significant difference in social preference for S1 and sociability index during the sociability test (Figure 5, H–J). However, there were significant increases in the time spent sniffing S2 and the social novelty index in MS^GAD67^-eNpHR-CKO mice during the social novelty test (Figure 5, K–M). Meanwhile, the firing rate of MS^GABA^ neurons in MS^GAD67^-eNpHR-CKO mice decreased significantly after 1 hour of yellow laser stimulation (Supplemental Figure 7, A–C). Following the repetitive yellow laser stimulation, MS^GAD67^-eGFP-CKO control mice showed no noticeable changes in sociability and social memory (Supplemental Figure 7, D–G). These data suggest that the inactivation of MS^GABA^ neurons increases NREM sleep and ameliorates social memory deficits in *Nlg3*-CKO mice, indicating that dual-functioning MS^GABA^ neurons regulate both social memory and sleep.

### MS^GABA^ neurons project to both POA and CA2.

**Figure 6:**
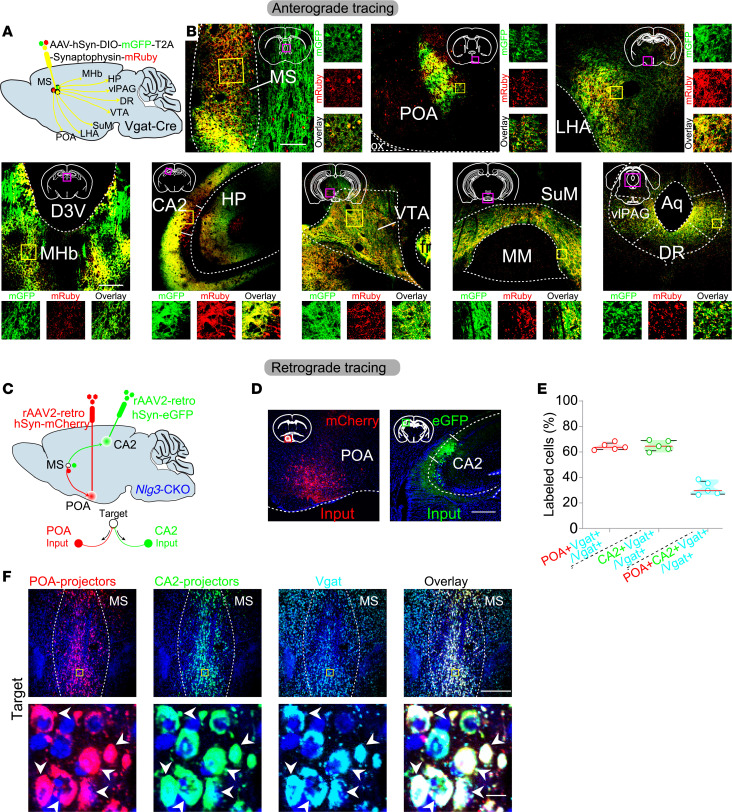
MS^GABA^ neurons project to both the POA and CA2. (**A**) Schematic drawing showing the axonal distributions of MS^GABA^ neurons in *Vgat*-Cre mice. MHb, medial habenula; HP, hippocampus; vlPAG, ventrolateral periaqueductal gray; DR, dorsal raphe; VTA, ventral tegmental area; SuM, supramammillary region; LHA, lateral hypothalamus area; POA, preoptic area. (**B**) Viral expression of mGFP and mRuby in MS^GABA^ neurons of *Vgat*-Cre mouse. MS^GABA^ neurons send projections to a variety of brain regions. Scale bar: 200 μm. ox, optic chiasm; 3V, 3rd ventricle; Aq, cerebral aqueduct; fr, fasciculus retroflexus. (**C**) Schematic of viral injection for simultaneous retrograde tracing from the POA and CA2 in *Nlg3*-CKO mice. (**D**) Representative images displaying retro-AAV injection sites in the POA (red) and CA2 (green). Scale bar: 200 μm. (**E**) Quantification of the percentage of labeled cells in the MS. Note that POA-projecting and CA2-projecting MS^GABA^ neurons are partially overlapping. *n* = 5 mice. POA+, POA-projecting cells; CA2+, CA2-projecting cells; Vgat+, MS^GABA^ neurons expressing Vgat. (**F**) Fluorescence images of MS showing the retrograde-labeled POA-projecting cells (in red), CA2-projecting cells (in green), and Vgat (in cyan) neurons. Scale bars: 300 μm (top) and 20 μm (bottom).

We next investigated the downstream projections that regulate 2 distinctly different physiological modalities. We conducted anterograde tracing of MS^GABA^ neurons in *Nlg3*-CKO mice and identified a series of downstream brain regions that are known to regulate either sleep/wakefulness or social memory. These regions include the preoptic area (POA) (21–23), lateral hypothalamus area (LHA) (24–26), medial habenula (MHb) (27), hippocampal CA2 (28–33), ventral tegmental area (VTA) (6, 34, 35), ventrolateral periaqueductal gray (vlPAG) (36), supramammillary region (SuM) (37–39), and dorsal raphe (DR) (40) (Figure 6, A and B, and Supplemental Figure 8, A and B). It has been postulated that the activation of GABAergic neurons could potentially reduce sleep by inhibiting sleep-promoting neurons (41–43), and impair social memory through inactivation of social memory–encoding neurons (44, 45).

It is well known that the POA is enriched in sleep-promoting neurons (21–23), while the CA2 contains the neurons that encode social memory (28–33), among the downstream projection regions of MS^GABA^ neurons. We injected rAAV2 retroviruses into the POA and CA2 to express mCherry and eGFP, respectively, in projection fibers. This procedure allowed for the retrograde labeling of projection neurons in the MS of *Nlg3*-CKO mice (46) (Figure 6, C–F). Notably, the overlap between mCherry-MS^GABA^ neurons and eGFP-MS^GABA^ neurons was approximately 30% (31.53% ± 2.35%), suggesting a portion of MS^GABA^ neurons divergently projected to both the POA and CA2. What are the roles of downstream targets of the POA and CA2 in regulating sleep and social memory?

### Inhibition of CA2 or POA neurons in C57BL/6J mice innervated by MS^GABA^ neurons selectively impairs social memory or reduces sleep.

**Figure 7:**
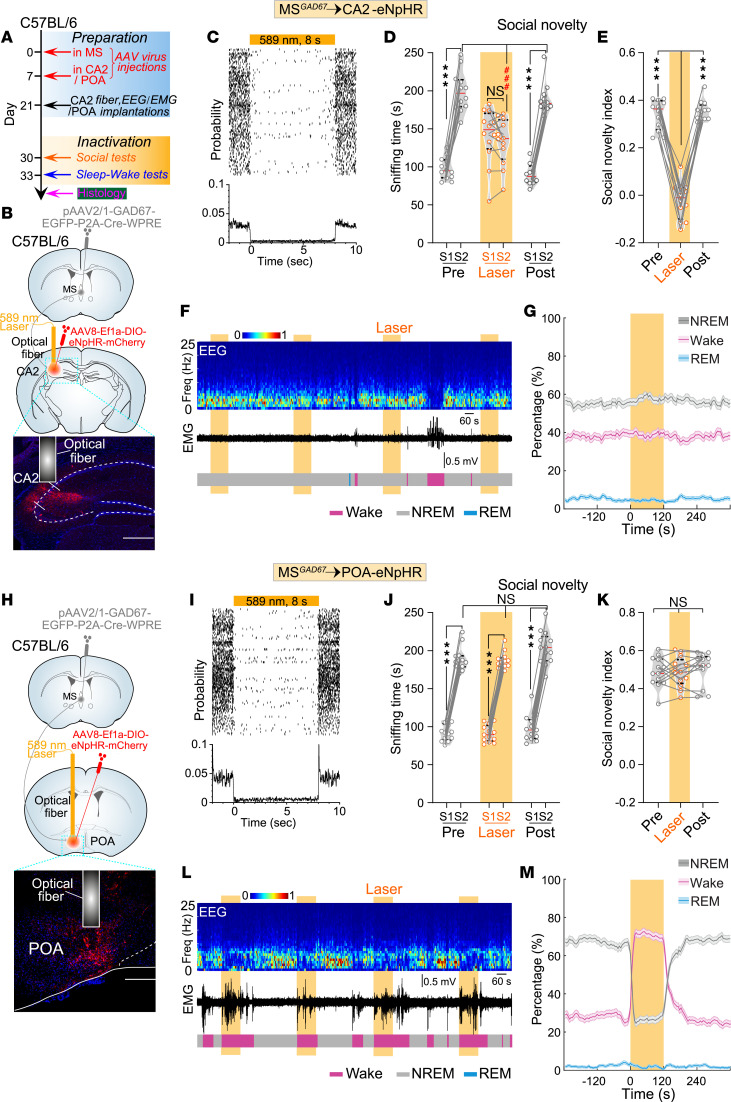
Inhibiting MS^GABA^-innervated CA2 or POA neurons selectively impairs social memory or reduces sleep, respectively, in C57BL/6J mice. (**A**) Schematic of the experimental procedure. (**B**) Schematic for labeling MS^GABA^→CA2 neurons in C57BL/6J mice. (**C**) Raster plot (top) and peristimulus time histogram (bottom) of a CA2 neuron innervated by MS^GABA^ neurons. (**D** and **E**) Quantification of sniffing time (**D**, *n* = 12 mice, *F*_[2,__33]_ = 128.8) and social novelty index (**E**, *F*_[1.84,__20.23]_ = 175.1) by inactivation of MS^GABA^-innervated CA2 neurons in C57BL/6J mice during the social novelty test. (**F**) Representative EEG spectrogram (top), relative EMG trace (middle), and brain states (bottom) from a C57BL/6J mouse. Yellow stripe indicates laser stimulation (589 nm, 8 seconds on/2 seconds off, 120 seconds). (**G**) No significant change in NREM sleep (*P* = 0.192), wakefulness (*P* = 0.349), and REM sleep (*P* = 0.376) during laser stimulation (*n* = 12 mice). Shading represents ±SEM. (**H**) Similar to **B**, but for labeling MS^GABA^→POA neurons. (**I**) Similar to **C**, but for an MS^GABA^-innervated POA neuron. (**J** and **K**) Quantification of sniffing time (**J**, *n* = 13 mice, *F*_[2,__36]_ = 3.865, *P* = 0.03) and social novelty index (**K**, *F*_[1.52,__18.25]_ = 0.643, *P* = 0.496) by inactivation of MS^GABA^-innervated POA neurons in C57BL/6J mice during the social novelty test. (**L**) Similar to **F**, but for inactivation of MS^GABA^-innervated POA neurons. (**M**) Optogenetic inactivation of MS^GABA^-innervated POA neurons (589 nm, yellow laser, 8 seconds on/2 seconds off, 120 seconds) significantly decreased in NREM sleep (*n* = 13 mice), increased in wakefulness, and did not affect REM sleep (*P* = 0.336). Shading represents ±SEM. ***P < 0.001; ^###^*P* < 0.001 by 2-way (**D** and **J**) or 1-way (**E** and **K**) repeated-measures ANOVA with Bonferroni’s post hoc test, or bootstrap test (**G** and **M**). NS, not significant.

To explore the functional roles of relevant downstream circuits, the conventional approach involves the activation of ChR2-expressing axon terminals in a specific target (47). However, this terminal activation has been shown to induce “antidromic spikes” and unwanted activation of collateral targets through antidromic stimulation (47). Therefore, to avoid “antidromic stimulation” and selectively inhibit CA2 or POA neurons innervated by MS^GABA^ neurons, we employed a dual virus approach and injected 2 separate vectors (Figure 7, A, B, and H). Specifically, we first injected anterograde trans-synaptic AAV (pAAV2/1-GAD67-EGFP-P2A-Cre-WPRE) into the MS of C57BL/6J mice, and subsequently injected another AAV (AAV8-Ef1a-DIO-eNpHR-mCherry) into either the CA2 or POA 1 week later (Figure 7, B and H). As a result, eNpHR-mCherry was selectively expressed in the CA2 or POA neurons that were innervated by MS^GABA^ neurons in C57BL/6J mice. Moreover, after injection of only AAV8-Ef1a-DIO-eNpHR-mCherry into the CA2 and POA we did not detect any eNpHR-mCherry in the CA2 and POA (Supplemental Figure 8, C and D).

One month after virus injection in C57BL/6J mice, the optogenetic inhibition (589 nm, 8 seconds on/2 seconds off) of CA2 neurons innervated by MS^GABA^ neurons did not have an impact on sociability (Figure 7C and Supplemental Figure 8, E and F), but significantly impaired social memory (Figure 7, D and E). However, this inhibition of CA2 neurons did not affect the sleep/wakefulness rhythm (Figure 7, F and G). In contrast, when the same optogenetic inhibition was applied to POA neurons innervated by MS^GABA^ neurons (Figure 7, H and I), we did not find a significant change in either sociability or social memory (Figure 7, J and K, and Supplemental Figure 8, G and H), but there was a notable decrease in NREM sleep and an increase in wakefulness (Figure 7, L and M). These collective findings demonstrate that the inhibition of the CA2 or POA neurons innervated by MS^GABA^ selectively impairs social memory or reduces sleep, respectively, in C57BL/6J mice.

### Activation of MS^GABA^-innervated CA2 or POA neurons selectively ameliorates social memory deficits or recovers lost sleep in Nlg3-CKO mice.

**Figure 8:**
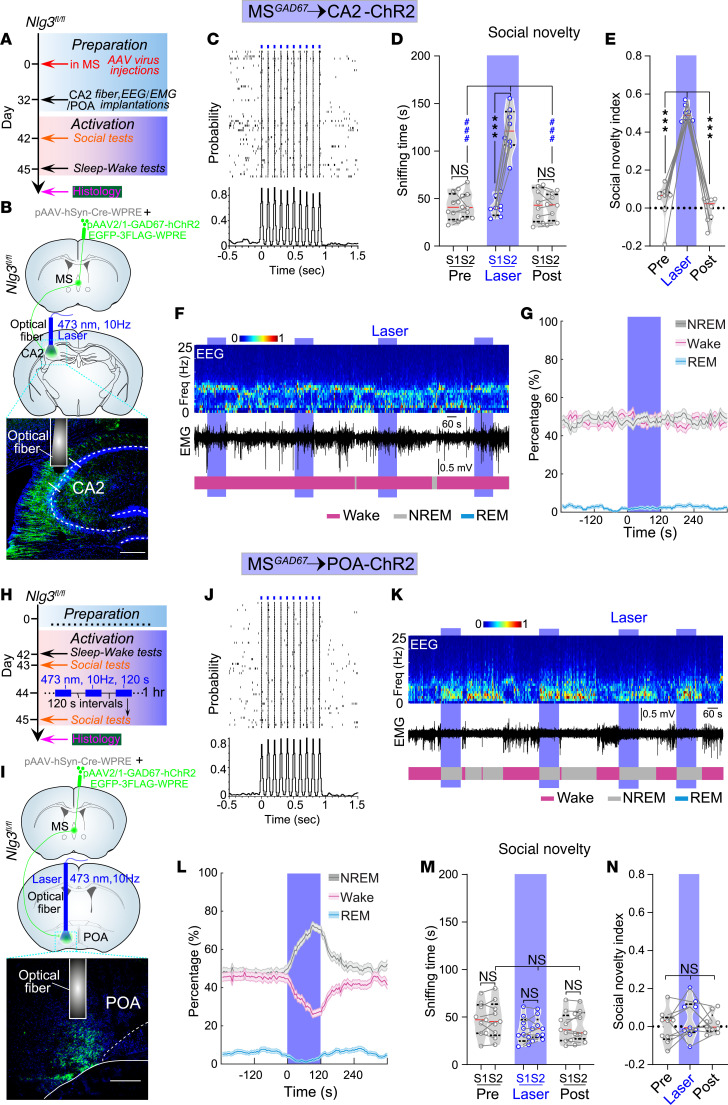
Activating MS^GABA^-innervated CA2 or POA neurons selectively ameliorates social memory deficits or recovers lost sleep, respectively, in *Nlg3*-CKO mice. (**A**) Schematic of the experimental procedure. (**B**) Schematic for labeling MS^GABA^→CA2 neurons in *Nlg3*-CKO mice. (**C**) Raster plot and peristimulus time histogram of a CA2 neuron innervated by MS^GABA^ neurons. (**D** and **E**) Quantification of sniffing time (**D**, *n* = 8 mice, *F*_[2,__21]_ = 141.3) and social novelty index (**E**, *F*_[1.45,__10.14]_ = 99.7) by activation of MS^GABA^-innervated CA2 neurons in *Nlg3*-CKO mice during the social novelty test. (**F**) Representative EEG spectrogram, relative EMG trace, and brain states from an *Nlg3*-CKO mouse. Blue stripe indicates laser stimulation (473 nm, 10 Hz, 120 seconds). (**G**) No significant change in NREM sleep (*n* = 8 mice, *P* = 0.446), wakefulness (*P* = 0.125), and REM sleep (*P* = 0.465) during laser stimulation. Shading represents ±SEM. (**H**) Similar to **A**, but with a repetitive photostimulation paradigm to activate POA neurons for 1 hour before social tests. (**I**) Similar to **B**, but with fiber implantation into the POA. (**J**) Similar to **C**, but for an MS^GABA^-innervated POA neuron. (**K** and **L**) Activation of MS^GABA^-innervated POA neurons (473 nm, 10 Hz, 120 seconds) significantly increased in NREM sleep (*n* = 9 mice), decreased in both wakefulness and REM sleep. Shading represents ±SEM. (**M** and **N**) Quantification of sniffing time (**M**, *n* = 9 mice, *F*_[2,__24]_ = 0.127, *P =* 0.881) and social novelty index (**N**, *F*_[1.95,__15.57]_ = 0.81, *P* = 0.46) by activation of MS^GABA^-innervated POA neurons in *Nlg3*-CKO mice during the social novelty test. ***P < 0.001; ^###^*P* < 0.001 by 2-way (**D** and **M**) or 1-way (**E** and **N**) repeated-measures ANOVA with Bonferroni’s post hoc test, or bootstrap test (**G** and **L**). NS, not significant.

Finally, we investigate whether social memory deficits and sleep loss in *Nlg3*-CKO mice could be rescued by activation of CA2 or POA neurons innervated by MS^GABA^ neurons. To this end, we created *Nlg3*-CKO mice by injecting pAAV-hSyn-Cre into the MS of *Nlg3^fl/fl^* mice (Figure 8, A, B, H, and I). Simultaneously, we labeled CA2 or POA neurons innervated by MS^GABA^ neurons with hChR2-EGFP by an injection of anterograde trans-synaptic AAV (pAAV2/1-GAD67-hChR2-EGFP-3FLAG-WPRE).

Six weeks after virus injections, we performed optical activation (473 nm, 10 Hz) of the CA2 neurons innervated by MS^GABA^ neurons. Optical activation of the CA2 neurons did not change sociability of *Nlg3*-CKO mice (Figure 8C and Supplemental Figure 8, I and J), but ameliorated social memory impairment (Figure 8, D and E). However, this activation of CA2 neurons did not affect the sleep/wakefulness rhythm (Figure 8, F and G). In contrast, when the same optogenetic stimulation was applied to POA neurons innervated by MS^GABA^ neurons (Figure 8, H–J), we detected a notable increase in NREM sleep and a decrease in wakefulness and REM sleep (Figure 8, K and L). To prevent dozing off or falling asleep in *Nlg3*-CKO mice during social tests, we employed a repetitive photostimulation paradigm to activate the POA neurons for 1 hour before social tests (Figure 8H). After application of blue laser stimulation to the POA neurons, *Nlg3*-CKO mice did not show any significant change in sociability (Supplemental Figure 8, K and M), and their social memory remained impaired (Figure 8, M and N). These findings demonstrate that activating MS^GABA^-innervated CA2 or POA neurons selectively ameliorates social memory deficits or recovers lost sleep in *Nlg3*-CKO mice, respectively.

Taken together, all these results indicate that MS^GABA^-innervated CA2 and POA neurons are 2 downstream targets that diverge the regulatory function of MS^GABA^ neurons toward social memory and sleep/wakefulness, respectively.

## Discussion

The core symptom of ASD (social deficits) and sleep disturbances are frequently encountered (48–50). Consistently, we found that CKO of autism-associated NLG3 in the MS not only impaired social memory, but also reduced sleep (Figure 1). The social memory deficits in *Nlg3*-CKO mice may be due to social anhedonia (Supplemental Figure 2, B and C). There were no significant differences in sociability and social novelty tests between female and male *Nlg3*-CKO mice (Supplemental Figure 9). Additionally, we observed that sleep deprivation caused the impairments in social memory and social isolation disrupted NREM sleep (Figure 4). However, it remains unknown whether there are shared cellular and neural circuit mechanisms underlying both social deficits and sleep disturbances. The MS is involved in both the modulation of social memory (10) and the regulation of sleep-wake (11). Notably, the MS is composed of cell types that are responsible for encoding social memory (10) or promoting wakefulness (11). The relationship between these 2 cell types remains elusive.

The main finding of this study is that the hyperactivity of MS^GABA^ neurons induced by *Nlg3* CKO in the MS caused social memory impairment and sleep disturbance through distinct downstream circuit targets. An additional finding is that the deletion of *Nlg3* in the MS, sleep deprivation, and social isolation led to a reduction in NLG3 expression and an increase in MS^GABA^ neuron activity (Figures 1, 2, and 4). Specifically, these neurons exhibited hyperactivity during either social avoidance or wakefulness in *Nlg3*-CKO mice (Figure 2). The activation of these neurons profoundly impaired social memory and reduced NREM sleep in C57BL/6J mice (Figure 3). Conversely, inactivation of them ameliorated social memory deficits and increased NREM sleep in *Nlg3*-CKO mice (Figure 5). Furthermore, in C57BL/6J mice, silencing MS^GABA^-innervated CA2 neurons selectively impaired social memory but not sleep, whereas inhibiting MS^GABA^-innervated POA neurons reduced NREM sleep without affecting social memory (Figure 7). In contrast, in *Nlg3*-CKO mice, activating MS^GABA^-innervated CA2 or POA neurons selectively ameliorated social memory deficits or recovered lost sleep, respectively (Figure 8). Thus, these results demonstrate that MS^GABA^ neurons impair social memory and disrupt sleep following *Nlg3* CKO in the MS, and achieves their notable specificity through their divergent downstream targets.

How does CKO of *Nlg3* in MS result in the hyperactivity of MS^GABA^ neurons? A pioneering study has shown an increase in inhibitory synaptic transmission in NLG3 R451C (Arg^451^→Cys^451^) mice (15). The increase in inhibitory synaptic strength in these mice could be attributed to the remaining NLG3 protein (~10% of total), suggesting a gain-of-function mutation. Furthermore, both NLG3 R451C and NLG3 knockout enhance GABAergic synaptic transmission in cholecystokinin (CCK) basket cell synapses in the hippocampus, which is attributed to the disruption of tonic endocannabinoid signaling at presynapses (51). In contrast, the deletion of NLG3 specifically in CA1 parvalbumin (PV) interneurons leads to an increase in glutamate release at presynapses by impairing presynaptic Group III metabotropic glutamate receptors (52). In our study, *Nlg3*-CKO in the MS reduced approximately 90% of the total NLG3 protein (Figure 1), resulting in the hyperactivity of MS^GABA^ neurons (Figure 2). Therefore, it is possible that the loss of tonic endocannabinoid signaling could account for the hyperactivity and further investigation is required to verify this. Furthermore, we consistently support the notion that the disruptions in the inhibitory/excitatory (I/E) balance contribute to the pathogenesis of ASD (53–58). The I/E imbalance results in abnormal oscillatory activity in different neuronal networks (55, 56), including the networks from MS to CA2 or POA. Eventually, this imbalance may also lead to the comorbidity between social memory impairments and sleep disturbances.

The relationship between social deficits and sleep disturbances is bidirectional, meaning that social deficits can disrupt sleep, and sleep disturbances can in turn impair social behaviors (59). This bidirectional relationship contributes to perpetuating and amplifying both sleep disturbances and social deficits (Figure 4). We subsequently investigated the downstream targets involved in this bidirectional relationship. Anterograde tracing of MS^GABA^ axons expressing eGFP showed that MS^GABA^ neurons projected to various brain regions that participate in the control of sleep-wakefulness and social behaviors, such as the POA (21–23), LHA (24–26), MHb (27), CA2 (28–33), VTA (6, 34, 35), vlPAG (36), SuM (37–39), and DR (40) (Figure 6). The POA region is known to contain various sleep-promoting cell types and plays a critical role in regulating sleep (21–23), and CA2 is involved in encoding social memory (28–33). Furthermore, retrograde tracing provided evidence that MS^GABA^ neurons simultaneously send monosynaptic inputs onto POA and CA2 neurons (Figure 6). Interestingly, in C57BL/6J mice, silencing CA2 neurons innervated by MS^GABA^ neurons selectively impaired social memory but not sleep, whereas inhibiting POA neurons innervated by MS^GABA^ neurons reduced NREM sleep without affecting social memory (Figure 7). In contrast, in *Nlg3*-CKO mice, activating MS^GABA^-innervated CA2 or POA neurons selectively ameliorated social memory deficits or recovered lost sleep, respectively (Figure 8). We speculate that CKO of sleep-wake behaviors in the MS results in the hyperactivity of MS^GABA^ neurons. Consequently, the hyperactivity of MS^GABA^ neurons could potentially trigger MS→CA2 pathways to block social memory and MS→POA pathways to reduce NREM sleep. These findings may explain why social deficits and sleep disturbance are mutually interacting, with each increasing the risk for the emergence and/or exacerbation of the other, thus leading to a vicious cycle.

Besides the MS→CA2 and MS→POA projections, other potential pathways may also mediate social memory and sleep-wakefulness. Therefore, further studies are necessary to investigate the possible roles of projections from the MS to other brain regions in inhibiting social memory and reducing sleep, thereby fully encompassing the integration of all these areas in the modulation of sleep and social memory.

Sleep disturbances have been found to exacerbate ASD symptomatology (2). Patients with comorbid ASD and sleep disturbances tend to exhibit poor socialization abilities and significantly impaired social skills compared with ASD patients without sleep problems (48–50). Given the challenges associated with implementing certain behavioral interventions (60) and the limited efficacy of some pharmacotherapies for the comorbidity between social deficits and sleep disturbances (61, 62), the development of effective therapies is urgently required. Notably, our findings have shown that inactivating MS^GABA^ neurons not only recovered social memory, but also increased NREM sleep (Figure 5). These findings present the potential for the development of therapeutic interventions focused on MS, such as deep brain stimulation (63, 64) or transcranial magnetic stimulation (65, 66). Alternatively, small molecule inhibitors that specifically target MS^GABA^ neurons can be designed and produced for treating the comorbidity.

## Methods

### Sex as a biological variable.

Sex was not the primary variable in this study so the primary analysis pooled data from both sexes. Secondary analysis, which included sex as an independent variable, is presented in Supplemental Figure 9. We summarize all the data (Figure 1, G–K, Figure 5, I–M, and Supplemental Figure 6, C–F before laser inactivation) in sociability and social novelty tests from *Nlg3*-CKO mice and divide it into 2 groups according to sex. There were no significant differences in sociability and social novelty tests between female and male *Nlg3*-CKO mice (Supplemental Figure 9).

### Animals.

The male and female *Nlg3^fl/fl^* mice (stock 015835) and *Vgat*-Cre mice (stock 028862) were procured from The Jackson Laboratory, while the male and female C57BL/6J mice were obtained from the Laboratory Animal Center at Xuzhou Medical University. The mice, aged 3–5 months, were housed in a controlled environment with a consistent temperature of 22°C ± 1°C, humidity of 50% ± 1%, and subjected to a 12-hour light/12-hour dark cycle (with light exposure from 7 am to 7 pm). They were provided with unrestricted access to food and water.

### Viruses.

pAAV-hSyn-Cre, AAV-GAD67-hChR2-eGFP, AAV2-GAD67-eNpHR-eGFP, pAAV2/1-GAD67-EGFP-P2A-Cre-WPRE, pAAV2/1-GAD67-hChR2-EGFP-3FLAG-WPRE, AAV8-Ef1a-DIO-eNpHR-mCherry, AAV2-CaMKIIα-eNpHR-mCherry, and pAAV-control were purchased from OBIO. rAAV2-retro-hSyn-mCherry and rAAV2-retro-hSyn-eGFP were purchased from Brain VTA. AAV-hSyn-DIO-mGFP-T2A-Synaptophysin-mRuby was purchased from Brain Case. All viral vectors were aliquoted and stored at –80°C until use.

### Surgery.

Prior to the surgery, *Nlg3^fl/f^* or C57BL/6J mice were administered 1% sodium pentobarbital (40 mg/kg, i.p.) for anesthesia and subsequently positioned in a stereotaxic frame (RWD Life Technology Co. Ltd.). To maintain a stable body temperature, a heating pad set at 37°C–38°C was utilized throughout the duration of the surgery. In order to prevent ocular dryness, ophthalmic ointment was applied to the mice’s eyes. Following the completion of the surgery, the mice were transferred to a cage equipped with a heating pad and were only returned to their original home cage once they had fully regained consciousness.

### EEG/EMG recording electrode, optrode, and optical fiber implantations.

For EEG implantation, 2 stainless steel screws were carefully inserted into specific coordinates on the skull (bregma: AP = –3.5 mm, ML = ±3.0 mm). To record EMG, 2 Teflon-coated annealed stainless steel wires were inserted into the musculature of the neck. Subsequently, the EEG and EMG electrodes were securely attached to a connector. Additionally, custom-made optrodes or optical fibers were implanted into specific brain regions (MS, POA, or CA2) at the same location where the virus was injected. The optrodes or optical fibers were affixed to the skull alongside EEG and EMG electrodes using dental cement. Following the surgical procedure, a 1-week recuperation period was provided for the mice prior to conducting the experiments.

### Virus injection.

AAV was injected using a syringe nanoliter infusion pump (ZS, Dichuang) and a 10 μL Hamilton syringe (~350 nL at a rate of 100 nL/min), with a 5-minute pause implemented to minimize backflow. For CKO of *Nlg3* in the MS, pAAV-hSyn-Cre was injected into the MS of *Nlg3^fl/fl^* mice (bregma: AP = +0.68 mm, ML = +0.55 mm, DV = –3.7 mm, with an 8° angle toward the midline). For optrode recording of MS^GABA^ neurons in both *Nlg3*-CKO and control mice, a mixture of AAVs (pAAV-hSyn-Cre + AAV-GAD67-hChR2-eGFP) and a mixture of AAVs (pAAV-control + AAV-GAD67-hChR2-eGFP) were injected into the MS of *Nlg3^fl/fl^* mice and control mice, respectively. Additionally, for optogenetic manipulation of MS^GABA^ neurons in both C57BL/6J and *Nlg3*-CKO mice, AAV2-GAD67-hChR2-eGFP and a mixture of AAVs (pAAV-hSyn-Cre and AAV2-GAD67-eNpHR-eGFP) were respectively injected into the MS of C57BL/6J and *Nlg3^fl/fl^* mice. For retrograde tracing, rAAV2-retro-hSyn-mCherry and rAAV2-retro-hSyn-eGFP were injected into the POA (bregma: AP = +0.14 mm, ML = +0.8 mm, DV = –5.2 mm) and CA2 (bregma: AP = –1.6 mm, ML = +1.7 mm, DV = –1.6 mm), respectively. To optogenetically inactivate POA and CA2 neurons innervated by MS^GABA^ neurons in C57BL/6J mice, we first injected pAAV2/1-GAD67-EGFP-P2A-Cre-WPRE into the MS, followed by the injection of AAV8-Ef1a-DIO-eNpHR-mCherry into the POA and CA2 one week later, respectively. For optogenetic activation of POA and CA2 neurons innervated by MS^GABA^ neurons in *Nlg3*-CKO mice, we injected a mixture of AAVs (pAAV-hSyn-Cre-WPRE and pAAV2/1-GAD67-hChR2-EGFP-3FLAG-WPRE) into the MS of *Nlg3^fl/fl^* mice. We applied erythromycin ointment locally to prevent infection.

### FISH.

Mice were administered sodium pentobarbital (100 mg/kg) to induce deep anesthesia, followed by transcardial perfusion with ice-cold 0.1 M phosphate-buffered saline (PBS) and subsequent perfusion with 4% paraformaldehyde (PFA) in PBS. The mouse brains were meticulously dissected from the skull and then immersed in 4% PFA at 4°C for 24 hours to ensure proper fixation. Subsequently, the fixed brains were subjected to dehydration in 30% sucrose in PBS at 4°C for 48 hours. Following embedding and freezing, the brains were sectioned into 20-μm (for FISH) or 50-μm coronal slices using a cryostat. The RNAscope in situ hybridization technique was executed in accordance with the guidelines provided by the manufacturer, Advanced Cell Diagnostics. Subsequently, the images of sections were captured using a confocal microscope operated by Zen2 software (LSM 880, Zeiss), and the resulting images were processed using Fiji-ImageJ v1.52a (https://imagej.net/software/fiji/downloads).

### Laser capture microdissection.

To evaluate *Nlg3* knockout efficiency in MS^GABA^ and VGLUT2 neurons from *Nlg3*-CKO mice, we first labeled these neurons by using different viruses (AAV2-GAD67-eNpHR-eGFP, AAV2-CaMKIIa-eNpHR-mCherry). The brain tissue was then sliced into 20-μm-thick coronal sections and affixed to microscope slides. Laser microdissection and laser pressure catapulting were performed to capture GABAergic neurons and VGLUT2 neurons in the MS of the tissue sections using a PALM MicroBeam laser microdissection system (Carl Zeiss). A total of 30 MS-GABA and VGLUT2 neurons from each *Nlg3*-CKO mouse were collected for analyses. An equivalent number of cells were also extracted from the MS of *Nlg3^fl/fl^* mice.

### RT-PCR.

RNA was extracted from laser capture microdissection–isolated brain tissue using the Single Cell Sequence Specific Amplification Kit (Vazyme, P621) in accordance with the manufacturer’s instructions. The kit allows for the amplification of transcriptomes from single cells or small amounts of RNA, and detects the gene expression levels between individual cells. Amplification primers for *Nlg3* (R: 5′-CCAGGAGCCCAACGAAGATT-3′, F: 5′-CCACTGTCTCGGATGTCTTCA-3′) and *Gapdh* (R: 5′-TGTGTCCGTCGTGGATCTGA-3′, F: 5′-TTGCTGTTGAAGTCGCAGGAG-3′) were mixed to create an Assay Pool with a final primer concentration of 0.1 μM. RT-PCR was conducted in a 5 μL volume with 1 μL cell samples, 0.5 μL Assay Pool at 0.1 μM, 0.1 μL RT/Taq enzyme, and 2.5 μL of 2× Reaction Mix. The RT-PCR conditions were as follows: reverse transcription at 50°C for 60 minutes; initial denaturation at 95°C for 3 minutes, followed by 20 cycles of denaturation at 95°C for 15 seconds, annealing, and extension at 60°C for 15 minutes. Then, the RT-PCR products were used for subsequent qPCR reactions or stored at –20°C. qPCR is commonly used for DNA detection and quantification. cDNA diluted from the RT-PCR products was used as the template for real-time PCR with the primers *Nlg3* (R: 5′-CCAGGAGCCCAACGAAGATT-3′, F: 5′-CCACTGTCTCGGATGTCTTCA-3′) and *Gapdh* (R: 5′-TGTGTCCGTCGTGGATCTGA-3′, F: 5′-TTGCTGTTGAAGTCGCAGGAG-3′). A QuantStudio 7 Flex Real-time PCR system (Thermo Fisher Scientific) was used. Each reaction mixture volume (10 μL) contained 2× ChamQ SYBR qPCR Master Mix (Vazyme, P311) at 5 μL, each primer at 0.25 μL (10 μM), 0.5 μL of template, and each 50× ROX Reference Dye 1 at 0.2 μL. The cycling conditions included an initial denaturation step at 95°C for 30 seconds, followed by 40 cycles of denaturation at 95°C for 10 seconds and annealing/elongation at 60°C for 30 seconds. Samples were run in triplicate and product specificity was confirmed by agarose gel electrophoresis of real-time qPCR products.

### Western blotting.

MS brain tissue was collected and rinsed with PBS solution (Biosharp, BL601A) in a crushed-ice environment. Subsequently, the tissue was subjected to high-speed ultrasonic centrifugation (13,523*g*, 15 minutes, 4°C) in an ice-water environment for 5 minutes, along with 1 mL of RIPA Lysis Buffer (Strong, Beyotime, P001B) and 10 μL of protease inhibitor PMSF (Beyotime, ST506-2). The protein concentration of the sample was measured using the Enhanced BCA Protein Assay Kit (Beyotime, P0009). The sample was diluted with Omni-Easy Protein Sample Loading Buffer (EpiZyme, LT101) and then boiled at 100°C for 10 minutes. Equal amounts of the diluted sample and 4 μL of PageRuler (Thermo Fisher Scientific) were loaded onto 10% SDS-PAGE gels (EpiZyme PG112) for electrophoresis. Following blocking of the membranes with a 5% nonfat milk solution, they were incubated overnight at 4°C with antibodies against NLG3 (1:1000; Sigma-Aldrich, SAB1410991) and β-actin (1:1000; ZSGB-BIO, TA-09). The membranes were then treated with an HRP-conjugated secondary antibody (Beyotime, A0208 and A0216) for 2 hours, followed by 3 washes with TBST. After rewashing with TBST, the Ultrasensitive Substrate Pro (MonPro, PW30701) was added drop-wise, and imaged using an Alliance Q9 (Tengming) with NineAlliance x9 software.

### Statistics.

For the optogenetic and behavioral experiments, the mice were assigned randomly to either the control group or the experimental group. The summarized data in the violin plots are presented as the median (indicated by the red line) along with the 25th and 75th percentiles (represented by the dash line). The error bars in the time courses and cell number denote the SEM (Figure 1, M–O, Figure 2C, and Figure 4, I, N–P, and U), while the error bars in the brain state transition probabilities indicate the 95% confidence intervals (Supplemental Figures 5, H and I, and Supplemental Figure 6, A and B). To evaluate significant interactions between the group and time, a 2-way ANOVA was employed (Figure 1, G, J, and P–R, Figure 2, K and M, Figure 4, C, F, and Q–S, Supplemental Figure 2, A, O, and R, and Supplemental Figure 4, B, E, and H). Briefly, the effect size was initially computed as the discrepancy between 2 groups at each time point. Subsequently, we performed 2-way ANOVA (group × time). After confirming the significance of the main effect of the group, Bonferroni’s multiple-comparison test was employed to determine the specific time points at which group differences were significant. Two-tailed Mann-Whitney test was performed to compare the differences between 2 independent groups when the sample distributions were not normally distributed and the sample sizes were small (Figure 1, D, E, H, and K, Figure 2, C, D, and N, Figure 4, D, G, I, and U, and Supplemental Figure 2, B–D, F–H, J–M, P, and S). Two-way repeated-measures ANOVA was used for paired data at different time points in the 2 distinct groups (Figure 3, F and I, Figure 5, I and L, Figure 7, D and J, Figure 8, D and M, Supplemental Figure 5, C and F, Supplemental Figure 6, C and E, Supplemental Figure 7, D and F, and Supplemental Figure 8, E, G, I, and K). One-way repeated-measures ANOVA was used for paired data at different time points in the same group (Figure 3, G and J, Figure 5, J and M, Figure 7, E and K, Figure 8, E and N, Supplemental Figure 5, D and G, Supplemental Figure 6, D and F–H, Supplemental Figure 7, E and G, and Supplemental Figure 8, F, H, J, and M). A *P* value of less than 0.05 was considered significant. Statistical analyses were performed using GraphPad Prism 8.0., SigmaPlot 14.0, MATLAB 2017 (MathWorks), and SPSS v22 (IBM). It is important to note that each experiment was conducted a minimum of 3 times, yielding consistent results throughout the study.

### Study approval.

The animal care and all experiments used in this study were approved by the Institutional Animal Care and Use Committee and the Office of Laboratory Animal Resources of Xuzhou Medical University (protocol 202207S001) under the Regulations for the Administration of Affairs Concerning Experimental Animals (1988) in China.

### Data availability.

The complete data set generated in this study is described and provided in this document, in the supplemental material, and the source data file. Values for all data points in graphs can be found in the supplemental Supporting Data Values file. Because of the size and highly diverse nature and formats, these raw data are available from the corresponding author upon request.

A more detailed description of the materials and methods used is provided in Supplemental Methods.

## Author contributions

HS, JLC, WX, and SA designed the research. HS, YS, PN, XL, YL, ZQ, JS, YW, MW, XK, and SA performed the research. HS, YS, PN, XL, YL, ZQ, and SA analyzed data. HS, JLC, WX, and SA wrote the manuscript. JLC, WX, and SA supervised the project. All authors read and discussed the manuscript. HS was listed first in the order of co–first authors because HS was responsible for all experimental designs, performed research, collected, analyzed, and interpreted data, performed statistical analysis, and drafted and revised the manuscript.

## Supplementary Material

Supplemental data

Unedited blot and gel images

Supporting data values

## Version 1 07/26/2024

In-Press Preview

## Version 2 10/01/2024

Electronic publication
