# Supplementary material for: Autism-associated neuroligin 3 deficiency in medial septum causes social deficits and sleep loss in mice
Source: J Clin Invest. 2024 Jul 26;134(19):e176770. doi: 10.1172/JCI176770 (PMC11444198; doi:10.1172/JCI176770)
Supplement: Supplemental data [file jci-134-176770-s080.pdf]

Autism-associated neuroligin-3 deficiency in medial septum causes social deficits and sleep loss in mice

Authors

Haiyan Sun[1,*], Yu Shen[1], Pengtao Ni[1], Xin Liu[1], Yan Li[1], Zhentong Qiu[1], Jiawen Su[1], Yihan Wang[1], Miao Wu[3], Xiangxi Kong[1], Jun-Li Cao[1,2,*], Wei Xie[3,*], and Shuming An [1,*]

Affiliations

[1] Jiangsu Province Key Laboratory of Anesthesiology & Jiangsu Province Key Laboratory of Anesthesia and Analgesia Application Technology, NMPA Key Laboratory for Research and Evaluation of Narcotic and Psychotropic Drugs, Xuzhou Medical University, Xuzhou 221004, China.

[2] Department of Anesthesiology, Affiliated Hospital of Xuzhou Medical University, Xuzhou 221006, China.

[3] The Key Laboratory of Developmental Genes and Human Disease, Ministry of Education, School of Life Science and Technology, Southeast University, Nanjing 210096, China

Authorship note: H.S. and Y.S. contributed equally to this work. S.A., W.X., J.L.C., and H.S. jointly supervised this work.

*Correspondence Author: Email: shumingan@foxmail.com; wei.xie@seu.edu.cn; caojl0310@aliyun.com; haiyansunsmile@foxmail.com.

Conflict of interest: The authors have declared that no conflict of interest exists.

Supplemental Methods

Behavioral tests

Sociability and social novelty tests (1)

The chamber apparatus (60 cm × 40 cm × 23 cm) for the social recognition test was made of opaque acrylic panels. Two cylindrical metal grid cups (8 cm in diameter and 10 cm in height) were placed on the left and right sides, with the middle chamber remaining empty. The experiment comprised a habituation trial, followed by a sociability test and a social novelty test. During the habituation trial, two empty grid cups were placed on either side of the chamber. Each subject-mouse was placed in the center of the apparatus, with its back to the experimenter, and allowed to explore freely for 5 minutes while habituating to the apparatus and empty cups.

During the sociability test, a stranger mouse (Stranger 1, S1) and an inanimate object were respectively placed under two grid cups and positioned symmetrically on the left and right of the apparatus. Each subject-mouse was positioned at the center of the apparatus and allowed to freely explore for 10 minutes.

During the social novelty test, the object was replaced with another novel mouse (Stranger 2, S2). The subject-mice were placed in the same position to explore for 10 minutes. The sniffing time was defined as each time the nose of the experimental mouse touched or came within 2 cm of the cup edge. The sniffing time was recorded and analyzed using the VisuTrack Animal Behavior Analysis system (XinRuan Co.Ltd, Shanghai). The sociability index on the training trail was calculated by the following equation ($Time_{O}$, the time sniffing the object; $Time_{S1}$, the time sniffing the stranger

mouse S1). Sociability index = (Time_{S1} - Time_{O})/(Time_{S1} + Time_{O}). The Social novelty index on the test trail was calculated by the following equation (Time_{S1}, the time sniffing the familiar mouse S1; Time_{S2}, the time sniffing another novel mouse S2). Social novelty index = (Time_{S2} - Time_{S1})/(Time_{S2} + Time_{S1}). Following each trial, the apparatus, grid cups, and objects were cleaned with 75% ethanol to remove any residual odors that may affect subsequent tests.

24 hours later, the subject-mice with optogenetic inhibition (yellow laser, 589 nm, 10 mW, 8 s on/2 s off) or activation (blue laser, 473 nm, 5 mW, 10 Hz) were repeated for sociability and social novelty tests. For 1h inhibition (Figure 5A) or activation (Figure 8H) experiments, 2 min yellow (8 s on/2 s off) or blue (10 Hz) laser-ON followed by 2 min laser-OFF was used. At the beginning of every experiment, laser output was tested to ensure that at least 10 mW (yellow laser) and 5 mW (blue laser) of power were delivered to the ends of the optic fiber. When conducting multiple tests (before, during and after laser manipulation), the mice used as the stranger and the novel mice should be changed regularly to avoid exhaustion or social fatigue of the stranger mice. For one subject-mouse in sequential tests (the sociability and social novelty tests), however, the stranger mouse (Stranger 1, S1) in the sociability and social novelty tests must be the same one and should not be changed.

Social interaction test

Social interaction was conducted in the home cage of the test mouse according to the previous description (2). Cagemates were temporarily transferred to a

new cage and the test mouse was kept in the home cage for 1-min habituation. After habituation, an intruder mouse (4-5 weeks of age, same sex and strain) was introduced to the home cage for the social interaction (20 minutes). The cumulative time spent on anogenital sniffing (snout toward the anogenital area of conspecific) and pursuit (runs after the other mouse, while the head of the following mouse is directed at the backside of the other mouse) was recorded, respectively.

Sucrose preference test

Each test mouse was placed into a new cage with two bottles of water and habituated for 2 days (1-hour per day). After 2-day habituation, the water in one bottle was replaced with 1% sucrose solution for 1-hour test. The consumed weights of 1% sucrose solution and water were recorded. Sucrose preference was calculated as follows: sucrose preference = sucrose consumption / (sucrose consumption + water consumption).

Open field test

The open field chamber apparatus was a square Plexiglas box that comprised of four walls (50 cm × 30 cm) and one open roof (50 cm × 50 cm). The center zone, measuring 25 cm × 25 cm, was defined as the area located in the middle of the chamber. Each mouse was placed in the center of the chamber, with its back to the experimenter, and allowed to explore for 10 min. The time spent in the center, the distance traveled in the center and the total distance traveled were recorded and analyzed using the

VisuTrack Animal Behavior Analysis system (XinRuan Co.Ltd, Shanghai). Before each mouse was tested, the apparatus was cleaned thoroughly with 75% ethanol.

Elevated plus maze test

The elevated plus maze apparatus consisted of two open arms (30 cm × 5 cm) without any walls and two enclosed arms (30 cm × 5 cm) with 15 cm-high walls on each side. These arms extended from a 5 cm × 5 cm central platform. The apparatus was secured to a stabilizing support 70 cm above the ground. Each mouse was placed on the central platform, facing an open arm, with its back to the experimenter, and allowed to explore for 5 min. The time spent in the open arms, the distance traveled in the open arms and the total distance traveled were recorded and analyzed using the VisuTrack Animal Behavior Analysis system (XinRuan Co.Ltd, Shanghai). Before each mouse was tested, the apparatus was cleaned thoroughly with 75% ethanol.

Novel object recognition test

The chamber apparatus (60 cm × 40 cm × 23 cm) for the novel object recognition test was made of opaque acrylic panels. The experiment comprised a habituation trial, followed by a training trail and a test trial. During the habituation trial, two clean, empty, inverted pencil cups were placed into the chamber apparatus. Each mouse was placed in the center of the apparatus, with its back to the experimenter, and allowed to explore freely for 5 minutes while habituating to the apparatus and empty cups. During the training trial, two similar objects were placed under two grid cups and positioned symmetrically on the left and right of the apparatus. Each mouse was positioned at the center of the apparatus and permitted to investigate two indistinguishable objects

(object 1, O1 and object 2, O2) for 5 minutes. On the test trial, the familiar O1 or O2 was replaced with a new object (N). The mice were placed in the same position to explore for 5 minutes. The sniffing time was defined as each time the nose of the experimental mouse touched or came within 2 cm of the cup edge. The time spent sniffing objects was recorded and analyzed using the VisuTrack Animal Behavior Analysis system (XinRuan Co.Ltd, Shanghai). The discrimination score on the training trail was calculated by the following equation ($Time_{O1}$, the time sniffing object1; $Time_{O2}$, the time sniffing object2). Discrimination score = ($Time_{O2}$- $Time_{O1}$)/($Time_{O2}$+ $Time_{O1}$). The discrimination score on the test trail was calculated by the following equation ($Time_{O1/2}$, the time sniffing object1 or object2; $Time_{N}$, the time sniffing a new object). Discrimination score = ($Time_{N}$- $Time_{O1/2}$)/($Time_{N}$+ $Time_{O1/2}$). Following each trial, the apparatus, grid cups, and objects were cleaned with 75% ethanol to remove any residual odors that may affect subsequent tests.

Brain states recording

The experiment comprised habituation trials and recording trials. For habituations, mice were given a minimum of two days to acclimate to the recording chamber and the attachment of recording cables. During the recording process, EEG and EMG electrodes were connected to recording headstages via flexible recording cables. The EEG/EMG signals were subsequently amplified, filtered (0–500 Hz), digitized at a rate of 1,500 Hz, and recorded using a NeuroLego amplifier (Jiangsu Brain Medical Technology Co.ltd, Nanjing, China). The EEG signals underwent spectral analysis has been conducted using the fast Fourier transform (FFT). The sleep-wake states were automatically classified into wake, NREM, or REM sleep using MATLAB, following established criteria published in previous studies (3, 4). Specifically, wakefulness was determined by the presence of desynchronized EEG and elevated EMG activity. NREM

sleep was characterized as synchronized EEG, high-amplitude, low-frequency (0.1-4 Hz) activity, and low EMG activity. During REM sleep, a consistent theta rhythm (5-10 Hz) with minimal EMG activity was observed. Following habituation, C57BL/6J mice were subjected to 24 hr undisturbed EEG and EMG recording starting at light onset in Figure 1L-O. The analyzers were blind to any information regarding the animal's identity or the timing of laser stimulation.

Optrode recordings for extracellular single-unit activities in freely moving mice

To identify MS^{GABA} neurons in freely moving mice, optrodes were fabricated using a custom optrode mold (3, 4). The optrodes were composed of a 200 μm diameter optic fiber (Thorlabs, FT200-UMT) fused to six pairs of stereotrodes. Each stereotrode was constructed by twisting two platinum-10%-iridium wires (35 μm in diameter, California Fine Wire, CA, USA) with an impedance of ~250 kΩ. Similarly, each tetrode consisted of four platinum-10%-iridium wires twisted together. The screw was affixed to the optrode, allowing for vertical movement via a screw-driven mechanism. During the recording, the researchers recorded single-unit activities while simultaneously monitoring brain states using EEG and EMG. The unit signals were amplified and filtered within the frequency range of 0.3 Hz to 8 kHz. Subsequently, the signals were digitized at a rate of 25 kHz and acquired using a NeuroLego amplifier (Jiangsu Brain Medical Technology Co.ltd, Nanjing, China). The optrodes were incrementally advanced by 50 μm each, with a maximum advancement of 150 μm per day. Upon completion of the recordings, an electrolytic lesion was created to indicate the end of

the electrode tract by applying a current of 100 μA for a duration of 10 seconds through two electrodes.

To record MS^{GABA} neurons during object and social exploration (Figure 2F), we performed optrode recordings while the subject-mouse was approaching and avoiding a novel inanimate object, a novel mouse, the familiar mouse. Similar with the chamber apparatus (60 cm × 40 cm × 23 cm) in sociability and social novelty tests, but only with one metal grid cup (6 cm × 9 cm) placed on either the left or right side. Each subject-mouse was positioned at the center of the apparatus and allowed to freely explore for at least 30 minutes in each section. The trajectory of the mouse was video-tracked using the VisuTrack Animal Behavior Analysis system (XinRuan Co.Ltd, Shanghai). Approaching transition was defined as the mouse entered the "approach zone" from "avoidance zone" with nose orientation towards the wire cage. Conversely, the avoiding transition was identified as the mouse entered the "avoidance zone" from "approach zone". This process was recorded as a trail, respectively. There are 189 trails from 7 mice for approaching transition from "avoidance zone" to "approach zone", and for avoiding transition from "approach zone" to "avoidance zone" (Figure 2J). Moreover, the activity of MS^{GABA} neurons in both NLG3-CKO and control mice was recorded and compared when these mice in "approach zone" and "avoidance zone" (Figure 2K and Supplemental Figure 4A-F). When conducting multiple days, the mice used as the novel and familiar mice should be changed regularly to avoid exhaustion or social fatigue of the novel and familiar mice.

The spikes originating from individual neurons were isolated through cluster

analysis using Offline Sorter (https://plexon.com/products/offline-sorter/). The clustering process involved offline sorting of spikes based on their waveform energy, peak amplitudes, and the first 3 principal components of the spike waveform on each stereotrode channel. In order to be classified as single units, clusters were required to satisfy two specific criteria: (1) refractory period (2 ms) violations were less than 0.2% of all spikes, and (2) isolation distance, estimated as the distance from the center of the identified cluster to the nearest cluster based on the Mahalanobis distance, was more than 20. Only stable and well-isolated units were used for further analysis. Subsequent data analyses, such as comparison of average firing rates, firing rate histograms, and rastergrams were performed with Matlab (Mathworks, 2017) and NeuroExplorer software (v_5.0 & v_5.2).

The identification of MS^{GABA} neurons was conducted using established methods (3, 4). In brief, intermittent delivery of 10Hz laser pulse trains (duration 1ms/pulse) with a duration of 1 s per train and an interval of 90 s was employed. As depicted in Supplemental Figure 3, ChR2-expressing neurons were deemed to meet three specific criteria: (1) laser pulses reliably elicited spikes (> 0.7 for all units in our sample) with a short latency for the first spike (< 3 ms for all units in our sample), (2) spikes exhibited minimal jitter (< 3 ms for all units in our sample), and (3) the waveforms of the laser-evoked and spontaneous spikes were highly similar (correlation coefficient > 0.95).

Optogenetic manipulations

Prior to optogenetic manipulations, the mice underwent a habituation session in the testing environment. During the optical stimulations, a blue 473-nm or yellow 589-nm laser light was administered by the laser system (Shanghai Laser & Optics Century, China) through a 200 μm diameter optic fiber (Inper, Hangzhou, China). This optic fiber was connected to a waveform generator (NeuroStim, Jiangsu Brain Medical Technology Co.ltd, Nanjing, China). Specifically, for the optogenetic activation of MS^{GABA} neurons depicted in Figure 3, blue laser pulses with a pulse width of 473 nm, power of ~5 mW, and frequencies of 10 were administered in a random manner, following a uniform distribution within a time range of 4 to 10 minutes. Similarly, for the optogenetic inactivation of MS^{GABA} neurons in Figure 5C-G, yellow laser pulses with a pulse width of 589 nm, power of approximately 10 mW, an 8-second on/2-second off pattern, and a duration of 2 minutes were randomly delivered within the same time range. However, to avoid dozing off or falling asleep in MS^{GAD67}-eNpHR-CKO mice during social tests, we applied a repetitive yellow-laser-stimulation paradigm (589 nm, 8 s on/2 s off, 120 s) that inhibited MS^{GABA} neurons for 1 hour prior to the social tests (Figure 5A). Similarly, a repetitive photo-stimulation paradigm to activate the POA neurons for 1 hour was employed before social tests (Figure 8H). Furthermore, a bootstrap procedure was conducted to assess the potential impact of laser application on brain state. Moreover, we determined the disparity between the average probabilities during laser application and the preceding period of equal duration through each iteration of the bootstrap, and subsequently computed the 95% confidence interval for this distribution of differences.

Sleep deprivation protocol

C57/BL6J mice were subjected to gentle stimulations, including exposure to an enriched, novel environment, novel objects, or light tapping/shaking of the cages, to induce wakefulness. Direct contact with the mice was avoided to minimize stress. Sleep deprivation was conducted at ZT 0 for a duration of 6 hours during the daytime. To ensure complete wakefulness, EEG and EMG signals were closely monitored throughout this period. Gentle stimulations were applied to the mice only when the EEG and EMG signals indicated non-rapid eye movement (NREM) sleep. Mice were left undisturbed when they were spontaneously awake.

QUANTIFICATION AND STATISTICAL ANALYSIS

Brain state transition probabilities

The computation of the transition probability was conducted using a time bin of 60 seconds (3). In this process, all trials (n) in which the animal was in state X (where X represents wakefulness, NREM, or REM) in the preceding time bin (i-1) were selected. Among these n trials, the subset of trials (m) in which the animal transitioned into state Y (where Y represents wakefulness, NREM, or REM) in the current time bin (i) were then tallied. Subsequently, the transition probability for X→Y at time bin (i) was calculated as m/n. Furthermore, the bootstrap procedure was employed to determine the 95% confidence intervals (CI) for the transition probabilities of brain states. Specifically, for an experimental group of n mice, with mouse i comprising m_i

trials, we first repeatedly resampled the data by randomly drawing for each mouse m_i trials (random sampling with replacement). Subsequently, the mean probabilities for each brain state transition were recalculated across the n mice for 10,000 iterations. The lower and upper confidence intervals were then extracted from the distribution of the resampled mean values. Additionally, bootstrap analysis was employed to assess the significance of laser stimulation in modulating a specific brain state. Lastly, for each bootstrap iteration, the disparities between the average probabilities observed during laser stimulation and the preceding period of equal duration were computed.

References

1. Rein B, Ma K, and Yan Z. A standardized social preference protocol for measuring social deficits in mouse models of autism. *Nature protocols.* 2020;15(10):3464-77.
2. Pomrenze MB, Cardozo Pinto DF, Neumann PA, Llorach P, Tucciarone JM, Morishita W, et al. Modulation of 5-HT release by dynorphin mediates social deficits during opioid withdrawal. *Neuron.* 2022;110(24):4125-43.e6.
3. An S, Sun H, Wu M, Xie D, Hu SW, Ding HL, et al. Medial septum glutamatergic neurons control wakefulness through a septo-hypothalamic circuit. *Current biology : CB.* 2021;31(7):1379-92.e4.
4. Xu M, Chung S, Zhang S, Zhong P, Ma C, Chang WC, et al. Basal forebrain circuit for sleep-wake control. *Nat Neurosci.* 2015;18(11):1641-7.

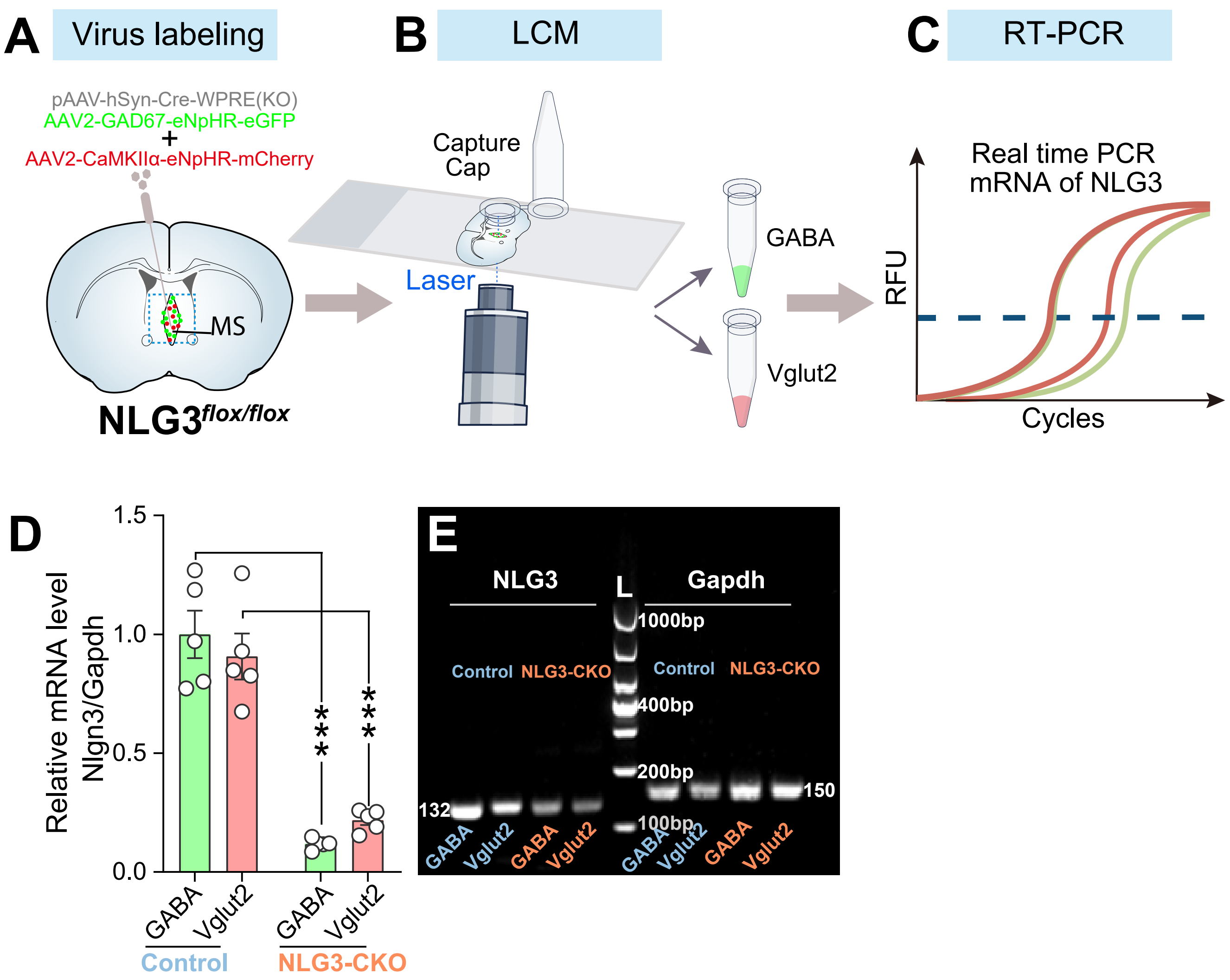
A
Virus labeling
pAAV-hSyn-Cre-WPRE(KO)
AAV2-GAD67-eNpHR-eGFP
+
AAV2-CaMKIIα-eNpHR-mCherry
MS
NLG3flox/flox
B
LCM
Capture
Cap
Laser
GABA
Vglut2
C
RT-PCR
Real time PCR
mRNA of NLG3
RFU
Cycles
D
Relative mRNA level
Nlgn3/Gapdh
1.5
1.0
0.5
0.0
***
***
GABA
Vglut2
GABA
Vglut2
Control
NLG3-CKO
E
NLG3
L
Gapdh
1000bp
Control
NLG3-CKO
Control
NLG3-CKO
400bp
200bp
132
150
100bp
GABA
Vglut2
GABA
Vglut2
GABA
Vglut2
GABA
Vglut2

Supplementary Fig. Legends

Supplementary Fig. 1 Decreased mRNA level of NLG3 in MS-GABA and Vglut2 neurons from NLG3-CKO mice.

(**A**) AAV2-GAD67-eNpHR-eGFP and AAV2-αCaMKII-eNpHR-mCherry were injected into MS to label GABA and Vglut2 neurons in NLG3-CKO and control mice, respectively. (**B**) MS-GABA and Vglut2 neurons were collected by laser capture microdissection (LCM) from NLG3-CKO and control mice. (**C**) A schematic of real-time reverse transcription-polymerase chain reaction (RT-PCR). (**D**) The relative mRNA level of NLG3 in MS-GABA and Vglut2 neurons from NLG3-CKO mice versus that from control mice ($F_{(3, 16)} = 41$, $P < 0.0001$, one-way ANOVA; control vs. NLG3-CKO, GABA: $P < 0.001$, Vglut2: $P < 0.001$; In control: GABA vs. Vglut2: $P = 0.678$, Bonferroni's multiple comparisons test). The data represent the mean ± sem, n=5 mice. (**E**) RT-PCR products (cDNA of NLG3:132 bp; Gapdh:150 pb). DNA Ladder (1000, 400, 200, and 100 bp) was used as a reference.

Supplementary Materials

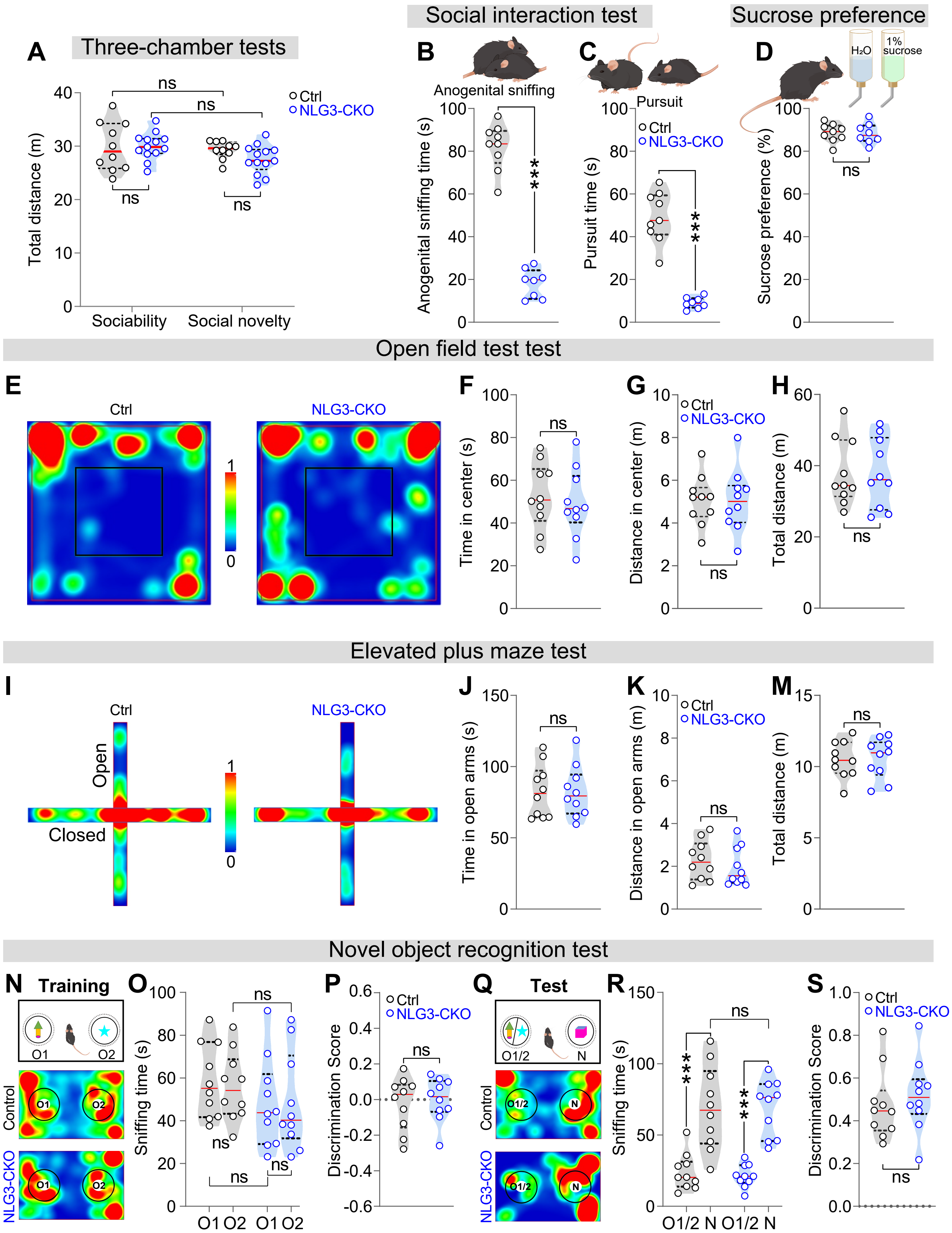

Supplementary Fig. 2 Conditional knockout of NLG3 in MS has no impact on sucrose preference, basal locomotion, anxiety-like behaviors, or cognitive ability for novel objects, but an impact on social interaction.

(**A**) Similar travel distance between control (*n* = 10 mice) and NLG3-CKO mice (*n* = 13 mice) in sociability and social novelty tests (Interaction, $F_{(1, 21)} = 1.342$, $P = 0.26$, two-way ANOVA; control vs. NLG3-CKO, Sociability: $P > 0.99$, Social novelty: $P = 0.31$; Bonferroni's multiple comparisons test). (**B-C**) NLG3-CKO mice spent significantly less time in anogenital sniffing (**B**; $P < 0.001$, Two-tailed Mann Whitney test) and pursuit (**C**; $P < 0.001$) than the control mice. (**D**) No significant difference was detected between NLG3-CKO (*n* = 8 mice) and control mice (*n* = 9 mice) in sucrose preference ($P = 0.743$, Two-tailed Mann-Whitney test). (**E-H**) Behavioral performance in the Open field task. (**E**) Representative heatmaps of the topographical time distribution in the open field test for a control and NLG3-CKO mouse. (**F-H**) No significant difference was detected between NLG3-CKO (*n* = 10 mice) and control mice (*n* = 10 mice) in the center time (**F**; $P = 0.481$, Two-tailed Mann Whitney test), center distance (**G**; $P = 0.853$), and total distance traveled (**H**; $P = 0.912$). (**I-M**) Behavioral performance in the elevated plus maze task. (**I**) Representative heatmaps of a control and NLG3-CKO mouse in elevated plus maze test. (**J-M**) No significant difference was detected between NLG3-CKO (*n* = 10 mice) and control mice (*n* = 10 mice) in the time spent in open arms (**J**; $P = 0.971$, Two-tailed Mann Whitney test), distance traveled in open arms (**K**; $P = 0.436$), and total distance traveled (M; $P = 0.853$). (**N-S**) Behavioral performance in the novel object recognition task. (**N**) Representative heatmaps of a control and NLG3-CKO mouse during the training phase. (**O-P**) NLG3-CKO (*n* = 10 mice) and control mice (*n* = 10 mice) spent a similar amount of time exploring two objects (**O**; Interaction, $F_{(1, 18)} = 0.226$, $P = 0.64$, Group, $F_{(1, 18)} = 1.18$, $P = 0.291$, two-way ANOVA; O1 vs. O2, control: $P = 0.809$, NLG3-CKO: $P = 0.996$; O1: control vs. NLG3-CKO, $P = 0.487$; O2: control vs. NLG3-CKO, $P = 0.809$; Bonferroni's multiple comparisons test) and displayed comparable discrimination scores (**P**; $P = 0.912$, Two-tailed Mann-Whitney test) during the training phase. (**Q**)

Representative heatmaps of a control and NLG3-CKO mouse during the test phase. (**R**) NLG3-CKO (*n* = 10 mice) and control mice (*n* = 10 mice) spent a similar amount of time exploring a novel object during the test phase (Interaction, $F_{(1, 18)} = 0.076$, $P = 0.786$, Group, $F_{(1, 18)} = 0.039$, $P = 0.850$, two-way ANOVA; N: control vs. NLG3-CKO, $P > 0.999$; O1/2: control vs. NLG3-CKO, $P > 0.999$; Time, $F_{(1, 18)} = 90.9$, $P < 0.001$, N vs. O1/2, control: $P < 0.001$, NLG3-CKO: $P < 0.001$; Bonferroni's multiple comparisons test). (**S**) No significant difference was observed between NLG3-CKO and control mice in the discrimination scores during the test phase ($P = 0.315$, Two-tailed Mann-Whitney test).

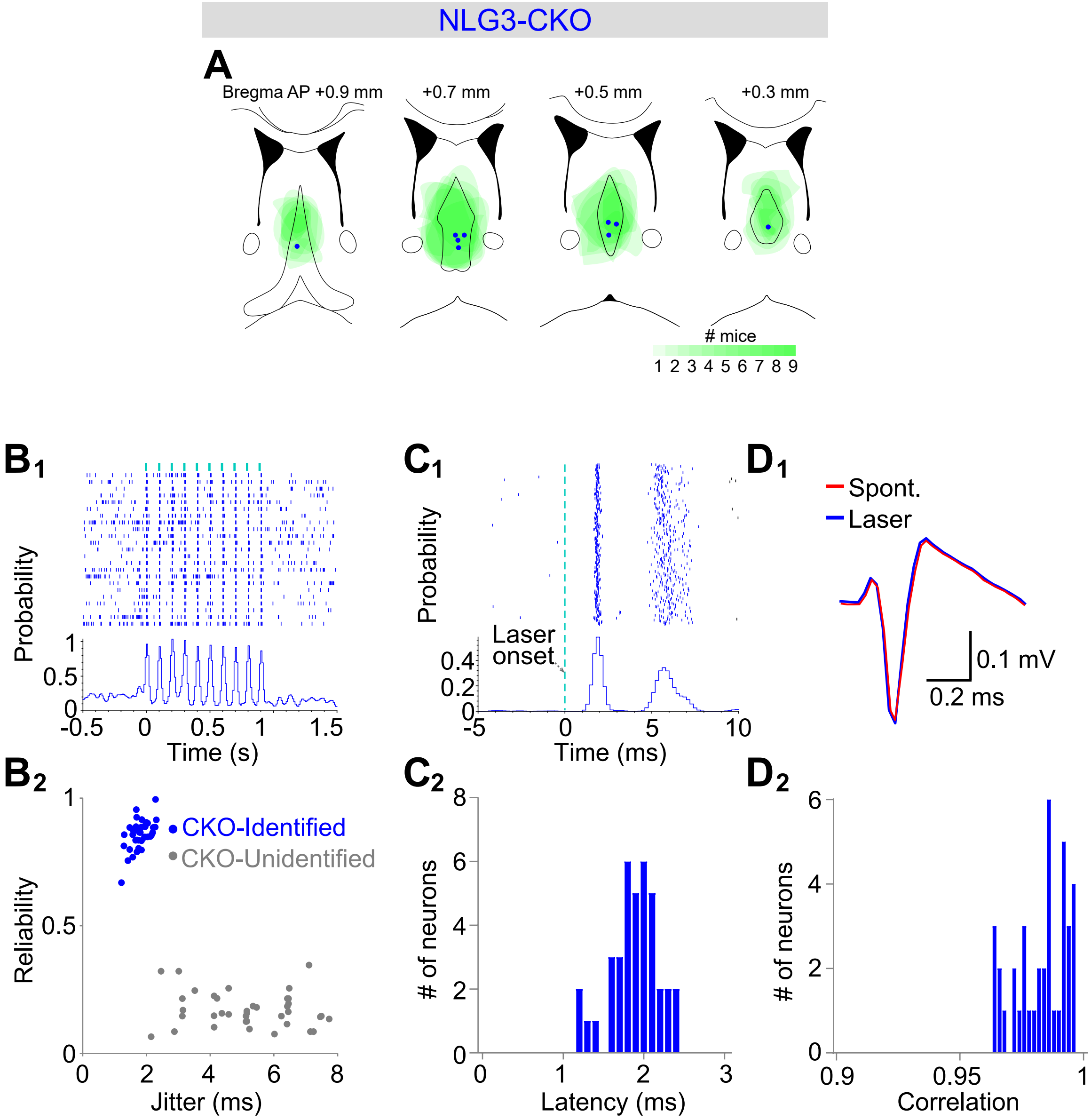
NLG3-CKO
A
Bregma AP +0.9 mm
+0.7 mm
+0.5 mm
+0.3 mm
mice
1 2 3 4 5 6 7 8 9
B1
Probability
Time (s)
C1
Probability
Laser onset
Time (ms)
D1
Spont.
Laser
0.1 mV
0.2 ms
B2
Reliability
CKO-Identified
CKO-Unidentified
Jitter (ms)
C2
# of neurons
Latency (ms)
D2
# of neurons
Correlation

Supplementary Fig. 3 Optogenetic identification of MS^{GABA} neurons

(**A**) The average distribution of ChR2-eGFP fluorescence expression in MS was shown for optrode recording in NLG3-CKO mice. For each mouse (n = 9 mice), 4 coronal brain sections were chosen to determine the spread of eGFP (from +0.9 mm to +0.3 mm along the rostrocaudal axis, where most of the virus expression was observed). Blue dots represent the positions of optrode tips. (**B$_1$**) A sample of peri-event raster plots (top) and peri-stimulus time histogram (PSTH, bottom) is presented, depicting the firing activity of a ChR2-tagged MS^{GABA} neuron in response to 10 Hz laser stimulation. Cyan ticks indicate laser pulses. (**B2**) The identified MS^{GABA} neurons (blue, n = 38 units from 7 mice) exhibit high reliability and low jitter, while unidentified neurons (gray, n = 38 units from 7 mice) demonstrate low reliability and diverse jitters. (**C1**) A sample of peri-event raster plots (top) and PSTH (bottom) for laser-evoked spikes of a ChR2-tagged MS^{GABA} neuron. The dashed line represents the time of laser onset. (**C2**) The plot illustrates the distribution of latencies, indicating that all ChR2-tagged MS^{GABA} neurons exhibit short latencies for laser-evoked spiking. (**D1**) The representative waveforms (averaged) of laser-evoked spikes (in blue) and spontaneous spikes (in red) are presented for an identified MS^{GABA} neuron. (**D2**) A distribution plot displaying high correlation coefficients between laser-evoked and spontaneous spike waveforms for all identified MS^{GABA} neuron.

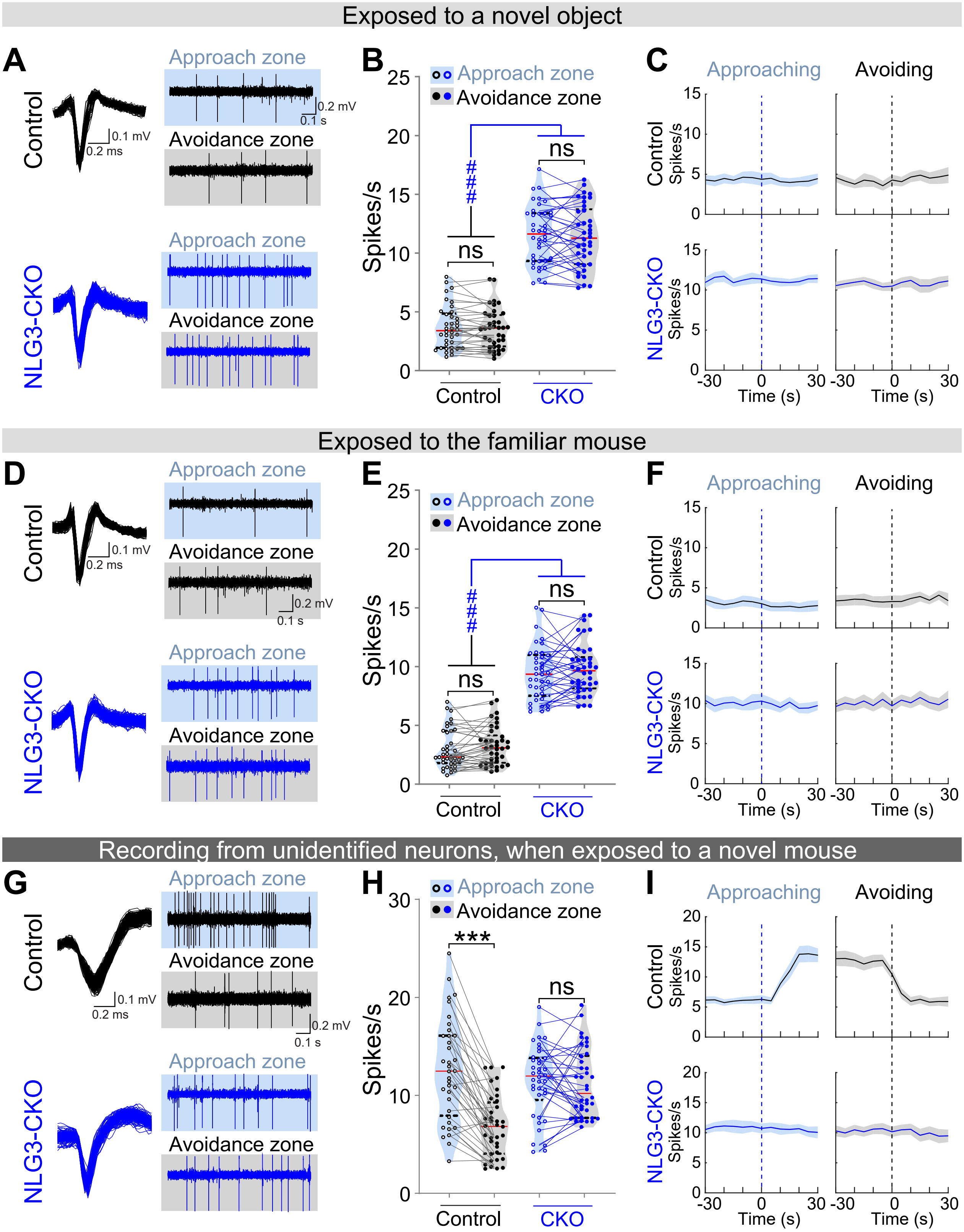

Exposed to a novel object
A
Control
NLG3-CKO
Approach zone
Avoidance zone
0.2 mV
0.1 s
0.1 mV
0.2 ms
B
Spikes/s
Approach zone
Avoidance zone
###
ns
Control
CKO
C
Approaching
Avoiding
Control Spikes/s
NLG3-CKO Spikes/s
Time (s)
Exposed to the familiar mouse
D
E
F
Recording from unidentified neurons, when exposed to a novel mouse
G
H
***
I

Supplementary Fig. 4 Optrode recording of MS^{GABA} neurons while the subject mice were exposed to a novel inanimate object and the familiar mouse.

(**A**) The waveforms show the spontaneous spikes in a ChR2-tagged MS^{GABA} neuron from control (black) and NLG3-CKO mice (blue). Right: Example recording of spontaneous spikes showing MS^{GABA} neuron firing rates in control and NLG3-CKO mice during the approach and avoidance of a novel object. (**B**) Violin plot displaying the individual firing rates of identified MS^{GABA} neurons from both NLG3-CKO mice (blue, n = 38 units from 7 mice) and control mice (black, n = 38 units from 7 mice) indicates no significant change in the firing rate while approaching and avoiding a novel object (Interaction, $F_{(1, 74)} = 0.0336$, $P = 0.855$; State, $F_{(1,74)} = 0.605$, $P = 0.439$, two-way ANOVA; approach vs. avoidance: NLG3-CKO, p = 0.998; control, p > 0.99; Bonferroni's multiple comparisons test). Moreover, the firing rate of MS^{GABA} neurons was significantly higher in NLG3-CKO mice than in control mice (Group, $F_{(1,74)}$ = 272.3, $P < 0.001$, two-way ANOVA; NLG3-CKO vs control: approach, $P < 0.001$; avoidance, $P < 0.001$; Bonferroni's multiple comparisons test). (**C**) Mean firing rates of identified MS^{GABA} neurons from both NLG3-CKO mice and control mice displaying no significant change in the firing rate while approaching and avoiding a novel object. Shading represents ± SEM. (**D-E**) Similar to (**A-C**), but the subject mice were exposed to the familiar mouse. (**E**) Violin plot displaying the individual firing rates of identified MS^{GABA} neurons from both NLG3-CKO mice (blue, n = 38 units from 7 mice) and control mice (black, n = 38 units from 7 mice) reveals no significant change in the firing rate while approaching and avoiding the familiar mouse (Interaction, $F_{(1, 74)} = 0.0386$, $P = 0.845$; State, $F_{(1,74)} = 2.535$, $P = 0.116$, two-way ANOVA; approach vs. avoidance: NLG3-CKO, $P = 0.42$; control, $P = 0.654$; Bonferroni's multiple comparisons test). Moreover, the firing rate of MS^{GABA} neurons was significantly higher in NLG3-CKO mice than in control mice (Group, $F_{(1,74)} = 257.4$, $P < 0.001$, two-way ANOVA; NLG3-CKO vs control: approach, $P < 0.001$; avoidance, $P < 0.001$; Bonferroni's multiple comparisons test). (**F**) Mean firing rates of identified MS^{GABA} neurons from both NLG3-CKO mice and control mice displaying no significant change in the firing rate

while approaching and avoiding the familiar mouse. Shading represents ± SEM. (**G-I**) Similar to (**A-C**), but recording from unidentified MS neurons, and the subject mice were exposed to a novel mouse. (**H**) Violin plot displaying the individual firing rates of unidentified MS neurons from both NLG3-CKO (blue, n = 38 units from 7 mice) and control mice (black, n = 36 units from 7 mice) during approaching and avoiding a novel mouse. The firing rate of unidentified MS neurons in control mice was significantly higher during approach of a novel mouse as compared to avoidance of a novel mouse, whereas this effect was not observed in NLG3-CKO mice (Interaction, $F_{(1, 72)} = 37.62$, $P < 0.001$; State, $F_{(1,72)} = 46.24$, $P < 0.001$, two-way ANOVA; approach vs. avoidance: Control, $P < 0.001$; NLG3-CKO, $P > 0.99$; Bonferroni's multiple comparisons test). (**I**) Mean firing rates of unidentified MS neurons from control mice showing increased firing rate during approaching the novel mouse and decreased firing rate during avoiding a novel mouse, but not from NLG3-CKO mice. Shading represents ± SEM.

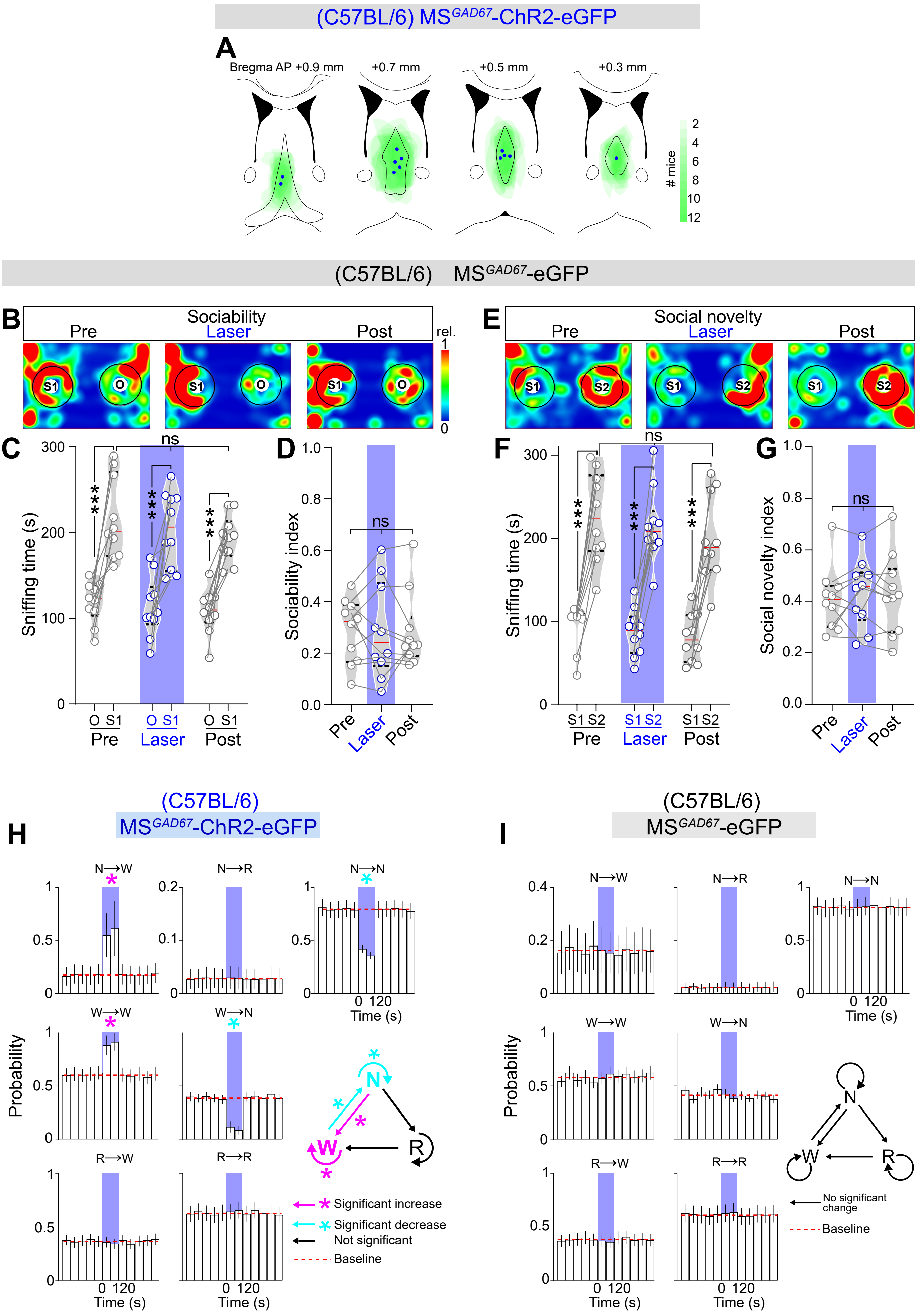

(C57BL/6) MSGAD67-ChR2-eGFP
A
Bregma AP +0.9 mm
+0.7 mm
+0.5 mm
+0.3 mm
mice
2
4
6
8
10
12
(C57BL/6) MSGAD67-eGFP
B
Sociability
Pre
Laser
Post
rel.
1
0
S1
O
E
Social novelty
S2
C
Sniffing time (s)
300
200
100
0
***
ns
O S1
Pre
Laser
Post
D
Sociability index
1.0
0.8
0.6
0.4
0.2
0.0
F
S1 S2
G
Social novelty index
(C57BL/6)
MSGAD67-ChR2-eGFP
H
N⟶W
N⟶R
N⟶N
W⟶W
W⟶N
R⟶W
R⟶R
Probability
Time (s)
0 120
N
W
R
Significant increase
Significant decrease
Not significant
Baseline
I
(C57BL/6)
MSGAD67-eGFP
No significant change
Baseline

Supplementary Fig. 5 Blue laser stimulation has no effect on the sociability and social memory in MS^{GAD67}-eGFP control mice.

(**A**) The average distribution of ChR2-eGFP fluorescence expression in MS was shown for optogenetic activation in MS^{GAD67}-ChR2-eGFP mice (*n* = 12 mice). For each mouse, 4 coronal brain sections were chosen to determine the spread of eGFP (from +0.9 mm to +0.3 mm along the rostrocaudal axis, where most of the virus expression was observed). Blue dots represent the positions of optic fiber tips. (**B**) Representative heatmaps of a MS^{GAD67}-eGFP control mouse illustrating the topographical time distribution in sociability tests, before, during, and after blue laser (473 nm, 10 Hz) stimulation. (**C-D**) MS^{GAD67}-eGFP mice showed no significant change in social preference for S1 (**C**; *n* = 10 mice, Interaction, $F_{(2, 27)} = 0.145$, $P = 0.866$; Treatment, $F_{(1.733, 31.20)} = 0.992$, *P = 0.372, Two-way Repeated measures ANOVA* test; S1: Pre vs. Laser, $P > 0.999$, Post vs. Laser, $P > 0.999$, Pre vs. Post, $P = 0.733$; O vs. S1: Pre, $P < 0.001$, Laser, $P < 0.001$, Post, $P < 0.001$; Bonferroni's multiple comparisons test) and sociability index (**D**; *n* = 10 mice, $F_{(1.93, 17.37)} = 0.005$, *P = 0.994, One-way Repeated measures ANOVA* test; Pre vs. Laser, $P > 0.999$, Post vs. Laser, $P > 0.999$, Pre vs. Post, $P > 0.999$; Bonferroni's multiple comparisons test) after optogenetic activation. (E-G) Similar to (B-D), but for MS^{GAD67}-eGFP in the social novelty test. Blue laser stimulation in MS^{GAD67}-eGFP mice did not affect the social preference for S2 (**F**; n = 10 mice, Interaction, $F_{(2, 27)} = 0.14$, $P = 0.87$; Treatment, $F_{(1.839, 33.10)} = 1.090$, *P = 0.343, Two-way Repeated measures ANOVA* test; S2: Pre vs. Laser, $P > 0.999$, Post vs. Laser, $P > 0.999$, Pre vs. Post, $P > 0.999$; S1 vs. S2: Pre, $P < 0.001$, Laser, $P < 0.001$, Post, $P < 0.001$; Bonferroni's multiple comparisons test) and social novelty index in MS^{GAD67}-eGFP mice (**G**; n = 10 mice, $F_{(1.97, 17.70)} = 0.268$, *P = 0.765, One-way Repeated measures ANOVA* test; Pre vs. Laser, $P > 0.999$, Post vs. Laser, $P > 0.999$, Pre vs. Post, $P > 0.999$; Bonferroni's multiple comparisons test). (**H-I**) Blue laser stimulation (10 Hz, 473 nm, 120 s) induced alterations in the transition probability between each pair of brain states in MS^{GAD67}-ChR2 mice (*n* = 12 mice), while no such changes were observed in MS^{GAD67}-eGFP mice (*n* = 10 mice). The diagram summarizes transitions that

exhibited significant increases (magenta), decreases (cyan), or remained unaffected (black) following laser stimulation. Magenta and cyan asterisk (*) indicate significant increase and decrease, respectively, in transition probability during laser stimulation compared to baseline (wake→wake, P = 0.021; wake→NREM, P = 0.022; NREM→wake, P = 0.015; NREM→NREM, P = 0.014; bootstrap). Bars represent transition probabilities within each 60 s period. Error bar, 95% CI (bootstrap).

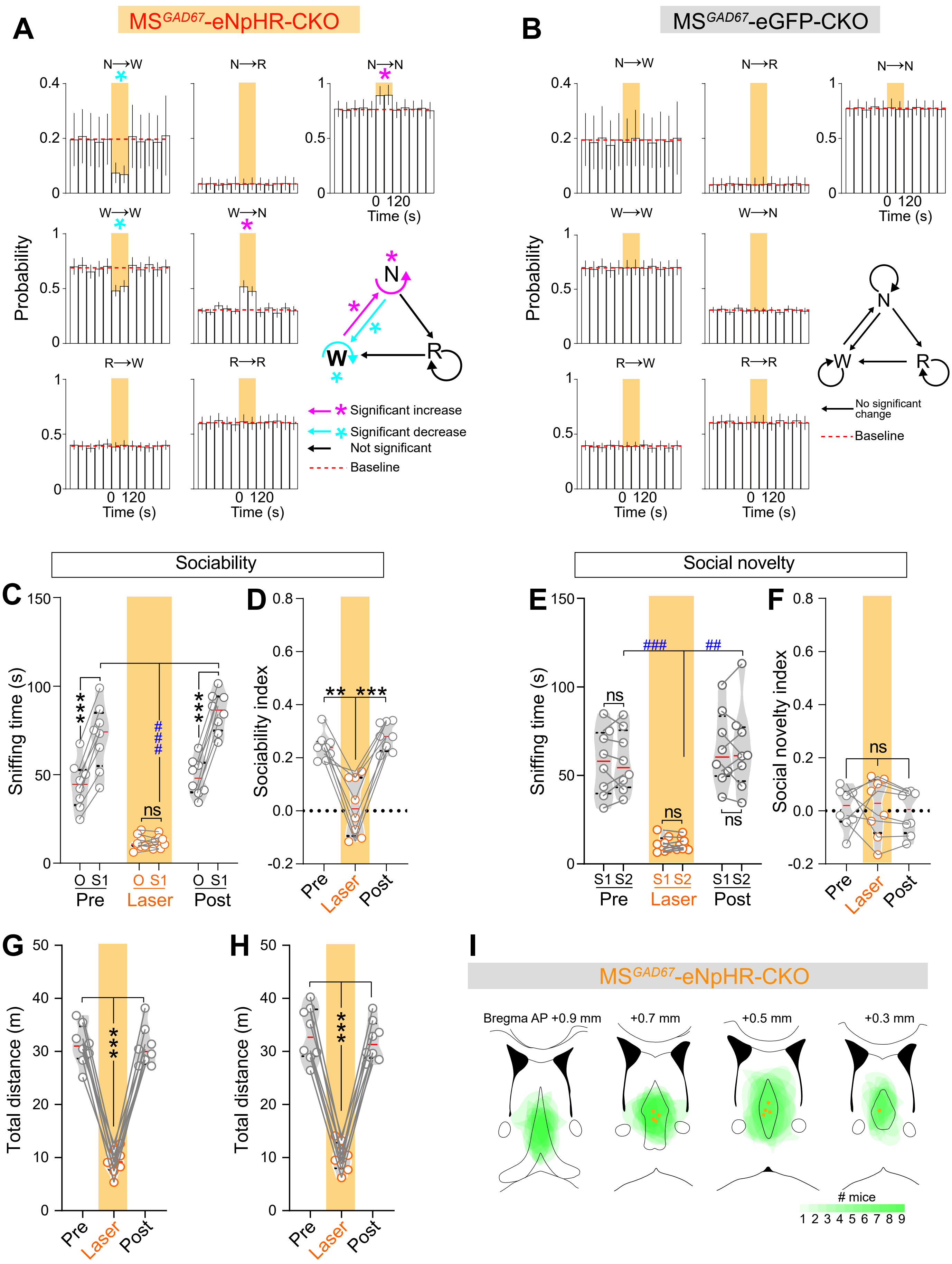

A
MSGAD67-eNpHR-CKO
B
MSGAD67-eGFP-CKO
N⟶W
N⟶R
N⟶N
W⟶W
W⟶N
R⟶W
R⟶R
Probability
Time (s)
Significant increase
Significant decrease
Not significant
Baseline
No significant change
Sociability
Social novelty
C
D
E
F
Sniffing time (s)
Sociability index
Social novelty index
O S1
S1 S2
Pre
Laser
Post
ns
G
H
I
Total distance (m)
Bregma AP +0.9 mm
+0.7 mm
+0.5 mm
+0.3 mm
mice
1 2 3 4 5 6 7 8 9

Supplementary Fig. 6 Yellow laser inactivation of MS^{GABA} neurons induces the changes of brain states transition in MS^{GAD67}-eNpHR-CKO mice, but not in MS^{GAD67}-eGFP-CKO control mice.

(**A**) Yellow laser inactivation of MS^{GABA} neurons (589 nm, 8 s on/2 s off,120 s) caused the changes of transition probability between each pair of brain states in MS^{GAD67}-eNpHR-CKO mice (n = 9 mice). The diagram summarizes transitions that exhibited significant increases (magenta), decreases (cyan), or remained unaffected (black) following laser stimulation. Magenta and cyan asterisk (*) indicate significant increase and decrease, respectively, in transition probability during laser stimulation compared to baseline (wake→wake, P = 0.021; wake→NREM, P = 0.023; NREM→wake, P = 0.032; NREM→NREM, P = 0.028; bootstrap). Bars represent of transition probabilities within each 60 s period. Error bar, 95% CI (bootstrap). (**B**) The same laser inactivation did not change the transition probability in MS^{GAD67}-eGFP-CKO control mice (n = 8 mice). ns, not significant. (**C-D**) In sociability test, silencing MS^{GABA} neurons in NLG3-CKO mice significantly decreased the sniffing time on both S1 and O (**C**; n = 8 mice, Interaction: $F_{(2, 21)}$ = 97.66*, $P < 0.001$; Treatment,* $F_{(1.28, 17.91)}$ = 98.51*, $P < 0.001$, Two-way Repeated measures ANOVA* test; S1: Pre vs. Laser, $P < 0.001$, Post vs. Laser, $P < 0.001$, Pre vs. Post, $P = 0.467$; O: Pre vs. Laser, $P = 0.0012$, Post vs. Laser, $P < 0.001$, Pre vs. Post, $P > 0.99$; Group: $F_{(1, 21)}$ = 372.2*, $P < 0.001$;*O vs. S1: Pre, $P < 0.001$, Laser, $P > 0.99$, Post, $P < 0.001$; Bonferroni's multiple comparisons test) and decreased sociability index (**D**; n = 8 mice, $F_{(1.531,10.72)}$ = 40.84*, $P < 0.001$, One-way Repeated measures ANOVA* test; Pre vs. Laser, $P = 0.0021$, Post vs. Laser, $P < 0.001$, Pre vs. Post, $P = 0.604$; Bonferroni's multiple comparisons test). (**E-F**) In social novelty test, silencing MS^{GABA} neurons in NLG3-CKO mice significantly decreased the sniffing time on both S1 and S2 (**E**; n = 8 mice, Interaction: $F_{(2, 21)}$ = 0.0095*, $P = 0.99$; Treatment,* $F_{(1.719, 24.06)}$ = 53.95*, $P < 0.001$, Two-way Repeated measures ANOVA* test; S1: Pre vs. Laser, $P = 0.0007$, Post vs. Laser, $P = 0.0004$, Pre vs. Post, $P > 0.99$; S2: Pre vs. Laser, $p = 0.0005$, Post vs. Laser, $P = 0.001$, Pre vs. Post, $P > 0.99$; Group: $F_{(1, 21)}$ = 0.019*, $P = 0.089$;* S1 vs. S2: Pre, $P > 0.99$, Laser, $P > 0.99$, Post, $P > 0.99$; Bonferroni's multiple

comparisons test), but had no significant effect on social novelty index (**F**; n = 8 mice, $F_{(1.059, 7.414)} = 0.149$, *P = 0.725, One-way Repeated measures ANOVA* test; Pre vs. Laser, *P > 0.99*, Post vs. Laser, *P = 0.378*, Pre vs. Post, *P > 0.99*; Bonferroni's multiple comparisons test). (**G-H**) Silencing MS^{GABA} neurons in NLG3-CKO mice significantly decreased the total distance travelled in sociability (**G**; n = 8 mice, $F_{(1.904, 13.33)} = 221.5$, *P < 0.001, One-way Repeated measures ANOVA* test; Pre vs. Laser, *P < 0.001*, Post vs. Laser, *P < 0.001*, Pre vs. Post, *P > 0.99*; Bonferroni's multiple comparisons test) and social novelty tests (**H**; n = 8 mice, $F_{(1.407, 9.85)} = 296.1$, *P < 0.001, One-way Repeated measures ANOVA* test; Pre vs. Laser, *p < 0.001*, Post vs. Laser, *P < 0.001*, Pre vs. Post, *P = 0.359*; Bonferroni's multiple comparisons test). (I) The average distribution of eNpHR-eGFP fluorescence expression in MS was shown for optogenetic inactivation in MS^{GAD67}-eNpHR-CKO mice (n = 9 mice). For each mouse, 4 coronal brain sections were chosen to determine the spread of eGFP (from +0.9 mm to +0.3 mm along the rostrocaudal axis, where most of the virus expression was observed). Orange dots represent the positions of optic fiber tips.

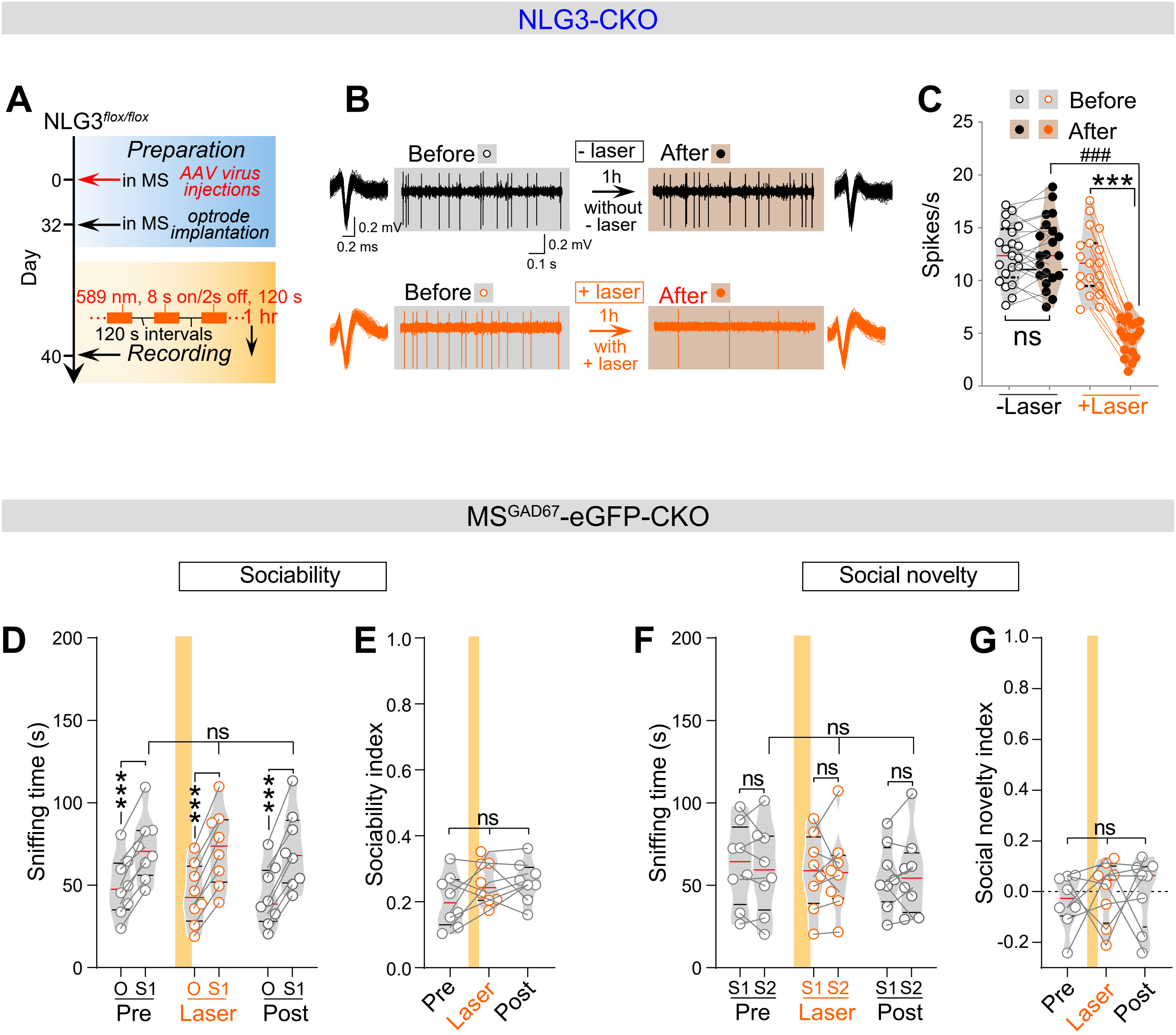
NLG3-CKO
A
NLG3flox/flox
Preparation
0
in MS
AAV virus injections
32
in MS
optrode implantation
Day
589 nm, 8 s on/2s off, 120 s
1 hr
120 s intervals
40
Recording
B
Before
- laser
1h
without
- laser
After
0.2 mV
0.2 ms
0.2 mV
0.1 s
Before
+ laser
1h
with
+ laser
After
C
Before
After
Spikes/s
###
***
ns
-Laser
+Laser
MSGAD67-eGFP-CKO
Sociability
Social novelty
D
Sniffing time (s)
***
***
***
ns
O S1
Pre
O S1
Laser
O S1
Post
E
Sociability index
ns
Pre
Laser
Post
F
Sniffing time (s)
ns
ns
ns
ns
S1 S2
Pre
S1 S2
Laser
S1 S2
Post
G
Social novelty index
ns
Pre
Laser
Post

Supplementary Fig. 7 One hour inactivation of MS^{GABA} neurons in NLG3-CKO mice reduced the firing rate of them, but had no effect on sociability and social memory in MS^{GAD67}-eGFP-CKO mice.

(**A**) Experimental scheme showing virus injections, optrode/fiber/EEG/EMG implantations, and social tests and optrode recording before, during, and after 1-hour inactivation of MS^{GABA} neurons in NLG3-CKO mice. (**B**) Example recording of spontaneous spikes showing MS^{GABA} neuron firing rates in NLG3-CKO mice with or without 1-hour laser inactivation. (**C**) Violin plot displaying the individual firing rates of MS^{GABA} neurons from NLG3-CKO mice with or without 1-hour laser inactivation. MS^{GABA} neurons in NLG3-CKO mice (n = 19 units from 4 mice) decreased the firing rate after 1-hour laser inactivation, whereas no effect was observed in control group (n = 21 units from 4 mice) without 1-hour laser inactivation (Interaction: $F_{(1,38)} = 107.9$, $P < 0.001$, Repeat measure two-way ANOVA; Time: $F_{(1,38)} = 97.21$, $P < 0.001$; Before vs. After: +Laser, $P < 0.001$; -Laser, $P > 0.99$; Group: $F_{(1,38)} = 31.35$, $P < 0.001$; +Laser vs. -Laser: After, $P < 0.001$; Before, $P = 0.687$, Bonferroni's multiple comparisons test). (**D-E**) MS^{GAD67}-eGFP-CKO mice showed no significant change in social preference for S1 (**D**; $n = 8$ mice, Interaction: $F_{(2, 21)} = 1.647$, $P = 0.217$; $F_{(1.429, 20.01)} = 0.165$, $P = 0.776$, *Two-way Repeated measures ANOVA* test; S1: Pre vs. Laser, $P > 0.999$, Post vs. Laser, $P > 0.999$, Pre vs. Post, $P > 0.999$; O vs. S1: Pre, $P < 0.001$, Laser, $P < 0.001$, Post, $P < 0.001$; Bonferroni's multiple comparisons test) and sociability index (**E**; $n = 8$ mice, $F_{(1.453, 17.37)} = 0.005$, $P = 0.994$, *One-way Repeated measures ANOVA* test; Pre vs. Laser, $P = 0.353$, Post vs. Laser, $P > 0.999$, Pre vs. Post, $P = 0.50$; Bonferroni's multiple comparisons test) after optogenetic activation. (**F-G**) Similar to (**D-E**), but for MS^{GAD67}-eGFP-CKO in the social novelty test. Blue laser stimulation in MS^{GAD67}-eGFP-CKO mice did not affect the social preference for S2 (**F**; $n = 8$ mice, Interaction: $F_{(2, 21)} = 1.619$, $P = 0.852$; $F_{(1.933, 27.06)} = 0.176$, $P = 0.832$, *Two-way Repeated measures ANOVA* test; S2: Pre vs. Laser, $P > 0.999$, Post vs. Laser, $P > 0.999$, Pre vs. Post, $P > 0.999$; S1 vs. S2: Pre, $P > 0.999$, Laser, $P > 0.999$, Post, $P > 0.999$; Bonferroni's multiple comparisons test) and social novelty index in MS^{GAD67}-eGFP-CKO mice (**G**;

n = 8 mice, $F_{(1.894, 13.26)} = 0.181$, *P = 0.825, One-way Repeated measures ANOVA* test; Pre vs. Laser, *P > 0.999*, Post vs. Laser, *P > 0.999*, Pre vs. Post, *P > 0.999*; Bonferroni's multiple comparisons test).

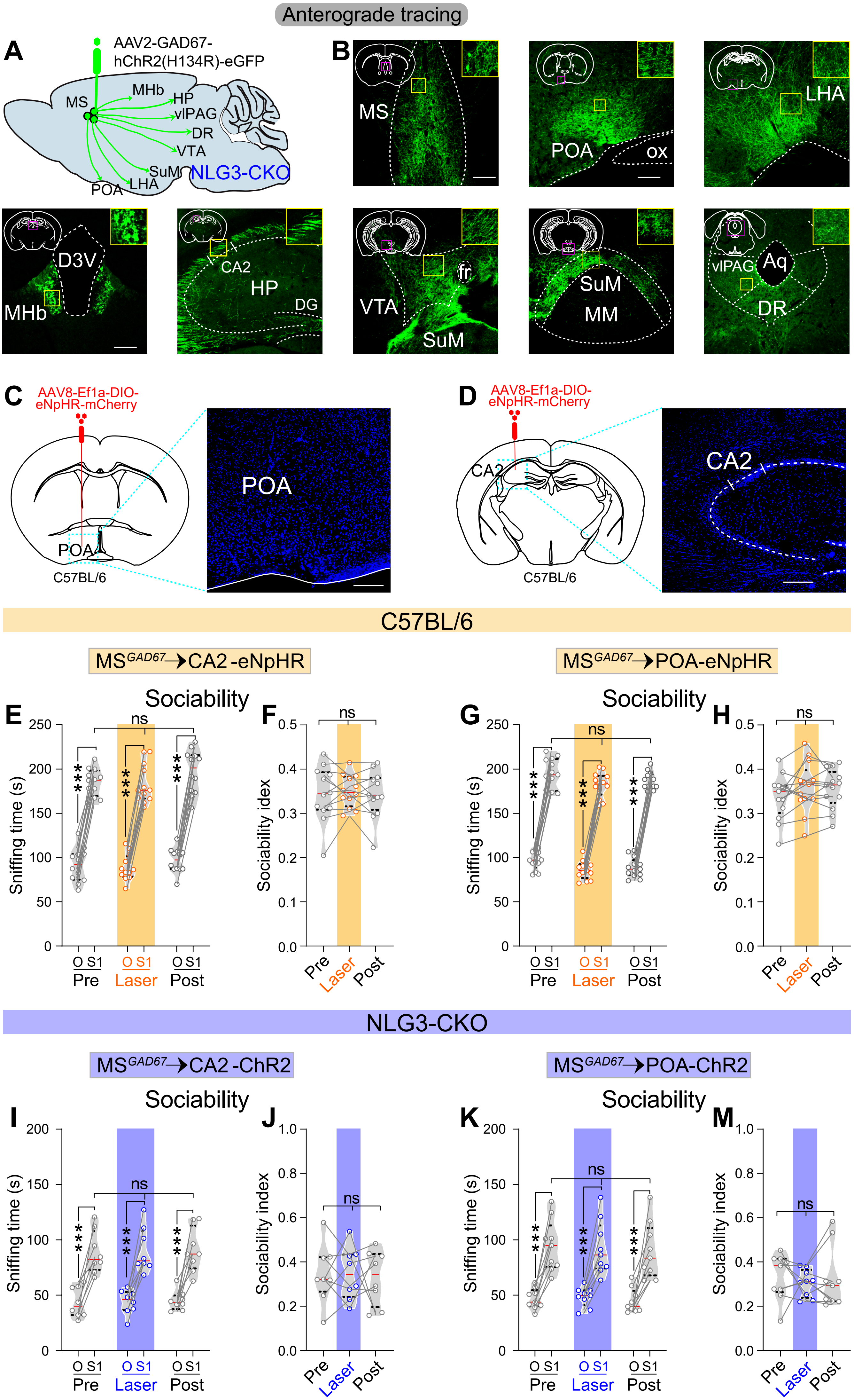

Anterograde tracing
A
AAV2-GAD67-hChR2(H134R)-eGFP
MS
MHb
HP
vlPAG
DR
VTA
SuM
LHA
POA
NLG3-CKO
B
MS
POA
ox
LHA
D3V
MHb
CA2
HP
DG
VTA
fr
SuM
MM
vlPAG
Aq
DR
C
AAV8-Ef1a-DIO-eNpHR-mCherry
POA
C57BL/6
D
AAV8-Ef1a-DIO-eNpHR-mCherry
CA2
C57BL/6
C57BL/6
MS^GAD67→CA2-eNpHR
MS^GAD67→POA-eNpHR
Sociability
E
Sniffing time (s)
O S1
Pre
Laser
Post
F
Sociability idex
G
H
NLG3-CKO
MS^GAD67→CA2-ChR2
MS^GAD67→POA-ChR2
I
J
Sociability index
K
M
ns
***

Supplementary Fig. 8 Neither the inhibition of CA2 neurons and POA neurons innervated by MS^{GABA} neurons in C57BL/6 mice, nor the activation of these neurons in NLG3-CKO mice has any effect on sociability.

(**A**) Schematic drawing showing the axonal distributions of MS^{GABA} neurons in NLG3-CKO mice. MHb, medial habenula; HP, hippocampus; vlPAG, ventrolateral periaqueductal gray; DR, dorsal raphe; VTA, ventral tegmental area; SuM, supramammillary region; LHA, lateral hypothalamus area; POA, preoptic area. (**B**) Viral expression of hChR2(H134R)-eGFP in MS^{GABA} neurons of NLG3-CKO mouse. MS^{GABA} neurons send projections to a variety of brain regions. Scale bar: 200 μm. ox, optic chiasm; 3V, 3rd ventricle; Aq, cerebral aqueduct; fr, fasciculus retroflexus. (**C**) Only injection of AAV8-Ef1a-DIO-eNpHR-mCherry into POA of C57BL/6 mice, without injection of pAAV2/1-GAD67-EGFP-P2A-Cre-WPRE into MS, resulted in the absence of mCherry expression in the POA. Right: a representative segment showing the overlap between DAPI and red-fluorescence in POA. Scale bar: 200 μm. (**D**) Similar to panel (**C**), but with the injection into CA2 in C57BL/6 mice. No mCherry expression in the CA2 (right). (**E** and **F**) Inhibition of CA2 neurons innervated by MS^{GABA} neurons in C57BL/6 mice did not significantly change the sniffing time for S1 (**E**; n = 12 mice, Interaction, $F_{(2, 33)} = 0.8727$, $P = 0.427$; Treatment, $F_{(1.681, 36.98)} = 2.491$, *$P = 0.105$, Two-way Repeated measures ANOVA* test; S1: Pre vs. Laser, $P > 0.999$, Post vs. Laser, $P = 0.687$, Pre vs. Post, $P = 0.627$; Group $F_{(1, 33)} = 1626$, $P < 0.001$; O vs. S1: Pre, $P < 0.001$, Laser, $P < 0.001$, Post, $P < 0.001$; Bonferroni's multiple comparisons test) and had no impact on sociability index (**F**; n = 12 mice, $F_{(1.874, 20.61)} = 0.24$, *$P = 0.775$, One-way Repeated measures ANOVA* test; Pre vs. Laser, $P > 0.999$, Post vs. Laser, $P > 0.999$, Pre vs. Post, $P > 0.999$; Bonferroni's multiple comparisons test). (**G** and **H**) Inhibition of POA neurons innervated by MS^{GABA} neurons in C57BL/6 mice did not significantly affect the sniffing time for S1 (**G**; n = 13 mice, Interaction, $F_{(2, 36)} = 0.045$, P= 0.956; *Two-way Repeated measures ANOVA* test; S1: Pre vs. Laser, $P = 0.140$, Post vs. Laser, $P > 0.999$, Pre vs. Post, $P = 0.636$; Group $F_{(1, 36)} = 1768$, $P < 0.001$; O vs. S1: Pre, $P < 0.001$, Laser, $P < 0.001$, Post, $P < 0.001$; Bonferroni's

multiple comparisons test) and had no impact on sociability index (**H**; *n* = 13 mice, $F_{(1.652, 19.83)}$ = 2.598, *P = 0.107, One-way Repeated measures ANOVA* test; Pre vs. Laser, *P = 0.213*, Post vs. Laser, *P > 0.999*, Pre vs. Post, *P = 0.432*; Bonferroni's multiple comparisons test). (**I** and **J**) Activation of CA2 neurons innervated by MS^{GABA} neurons in NLG3-CKO mice did not significantly change the sniffing time for S1 (**I**; *n* = 8 mice, Interaction, $F_{(2, 21)}$ = 0.016, *P*= 0.984; Treatment, $F_{(1.427, 19.97)}$ *= 0.045, P = 0.908, Two-way Repeated measures ANOVA* test; S1: Pre vs. Laser, *P > 0.999*, Post vs. Laser, *P > 0.999*, Pre vs. Post, *P > 0.999*; O vs. S1: Pre, *P < 0.001,* Laser, *P < 0.001*, Post, *P < 0.001;* Bonferroni's multiple comparisons test) and had no impact on sociability index (**J**; n = 8 mice, $F_{(1.721, 12.04)}$ = 0.049*, P = 0.933, One-way Repeated measures ANOVA* test; Pre vs. Laser, *P > 0.999*, Post vs. Laser, *P > 0.999*, Pre vs. Post, *P > 0.999*; Bonferroni's multiple comparisons test). (**K** and **M**) Activation of POA neurons innervated by MS^{GABA} neurons in NLG3-CKO mice did not significantly change the sniffing time for S1 (**K**; *n* = 9 mice, Interaction, $F_{(2, 24)}$ = 0.08, *P*= 0.923; Treatment, $F_{(1.352, 21.64)}$ = 0.218*, P = 0.718, Two-way Repeated measures ANOVA* test; S1: Pre vs. Laser, *P > 0.999*, Post vs. Laser, *P > 0.999*, Pre vs. Post, *P > 0.999*; O vs. S1: Pre, *P < 0.001,* Laser, *P < 0.001*, Post, *P < 0.001;* Bonferroni's multiple comparisons test) and had no impact on sociability index (**M**; *n* = 9 mice, $F_{(1.704, 13.63)}$ = 0.232*, P = 0.762, One-way Repeated measures ANOVA* test; Pre vs. Laser, *P > 0.999*, Post vs. Laser, *P > 0.999*, Pre vs. Post, *P > 0.999*; Bonferroni's multiple comparisons test).

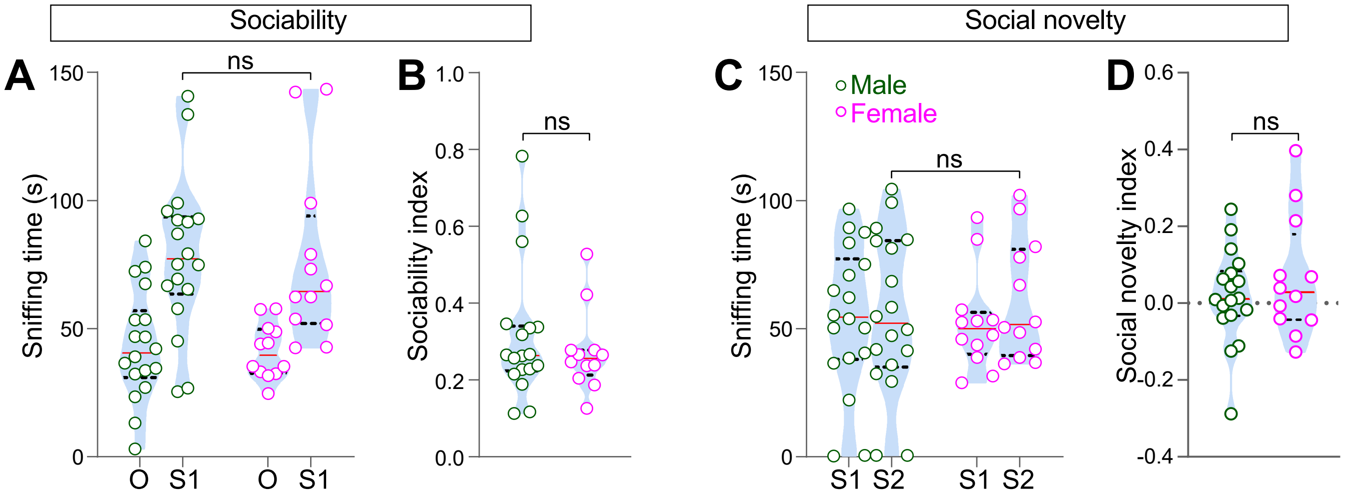

Sociability
Social novelty
A
B
C
D
ns
Sniffing time (s)
Sociability index
Social novelty index
Male
Female
O
S1
S2
150
100
50
0
1.0
0.8
0.6
0.4
0.2
0.0
-0.2
-0.4

Supplementary Fig. 9 No significant differences in sociability and social novelty tests between female and male NLG3-CKO mice. (A) In the sociability test, both male and female NLG3-CKO mice spent similar amounts of time sniffing the stranger mouse (male, n = 18 mice, female, n = 12 mice; Interaction, $F_{(1, 28)} < 0.001$, $P = 0.997$; *Two-way Repeated measures ANOVA* test). (B) The sociability index was comparable between male and female NLG3-CKO mice ($P = 0.70$, two-tailed Mann-Whitney test). (C-D) In the social novelty test, both male and female NLG3-CKO mice spent similar amounts of time sniffing S1 and S2 (C, Interaction, $F_{(1, 28)} = 1.614$, $P = 0.214$; *Two-way Repeated measures ANOVA* test), and exhibited similar social novelty index (D, $P = 0.787$, two-tailed Mann-Whitney test).
