# Supplementary figures and images for: Autism-associated neuroligin 3 deficiency in medial septum causes social deficits and sleep loss in mice
Source: J Clin Invest. 2024 Jul 26;134(19):e176770. doi: 10.1172/JCI176770 (PMC11444198; doi:10.1172/JCI176770)

## Full unedited blot for Figure 1E

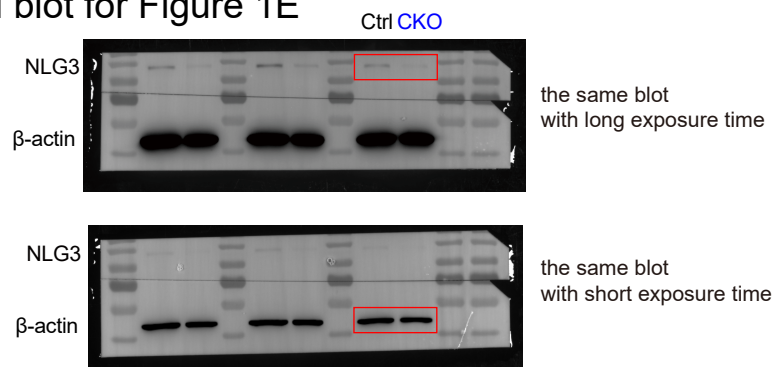

## Full unedited gel for Supplementary Fig. 1E

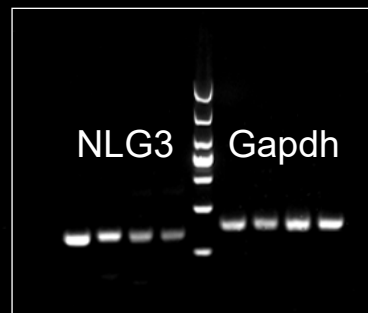

Supplement: Unedited blot and gel images [file jci-134-176770-s081.pdf]
